# Maternal serum retinol, 25(OH)D and 1,25(OH)_2_D concentrations during pregnancy and peak bone mass and trabecular bone score in adult offspring at 26-year follow-up

**DOI:** 10.1371/journal.pone.0222712

**Published:** 2019-09-26

**Authors:** Chandima N. D. Balasuriya, Tricia L. Larose, Mats P. Mosti, Kari Anne I. Evensen, Geir W. Jacobsen, Per M. Thorsby, Astrid Kamilla Stunes, Unni Syversen

**Affiliations:** 1 Department of Clinical and Molecular Medicine, Faculty of Medicine and Health Sciences, Norwegian University of Science and Technology, NTNU, Trondheim, Norway; 2 Department of Endocrinology, St. Olavs Hospital, Trondheim University Hospital, Trondheim, Norway; 3 K.G. Jebsen Center for Genetic Epidemiology, Department of Public Health and Nursing, Faculty of Medicine and Health Sciences, Norwegian University of Science and Technology, NTNU, Trondheim, Norway; 4 St. Olavs Hospital, Trondheim University Hospital, Trondheim, Norway; 5 Department of Public Health and Nursing, Norwegian University of Science and Technology, NTNU, Trondheim, Norway; 6 Department of Physiotherapy, Trondheim Municipality, Trondheim, Norway; 7 Hormone Laboratory, Department of Medical biochemistry, Oslo University Hospital Aker Hospital, Oslo, Norway; Texas A&M University College Station, UNITED STATES

## Abstract

**Background:**

Vitamin A and D deficiency is prevalent in pregnant women worldwide. Both vitamins are involved in fetal skeletal development. A positive association between maternal vitamin D levels and offspring bone mineral density (BMD) at adulthood has been observed. The impact of maternal vitamin A status in pregnancy on offspring peak bone mass remains unclear.

**Method and findings:**

Forty-one mother-child pairs were recruited from a population-based prospective cohort study in Trondheim, Norway, where pregnant women were followed from gestational week 17. Their term-born infants were followed from birth (1986–88). Regression analyses were performed for vitamin A (retinol), 25-hydroxyvitamin D [25(OH)D] and 1,25-dihydroxyvitamin D [1,25(OH)_2_D] in maternal serum (gestational weeks 17, 33, 37) and cord blood. Offspring BMD and spine trabecular bone score (TBS), a measure of bone quality, were analyzed by dual x-ray absorptiometry at 26 years. Average levels during pregnancy of retinol, 25(OH)D and 1,25(OH)_2_D were 1.66 (0.32) μmol/L, 59.0 (20.6) nmol/L, and 251.3 (62.4) pmol/L, respectively. 1,25(OH)_2_D levels were similar in those with 25(OH)D levels <30 and >75 nmol/L. After adjustment for maternal age, BMI, smoking, and education, and offspring birth weight, maternal serum retinol was positively associated with offspring spine BMD [mean change 30.8 (CI 7.6, 54.0) mg/cm^2^ per 0.2 μmol/L retinol], and with offspring TBS, although non-significant (p = 0.08). No associations were found between maternal 25(OH)D and 1,25(OH)_2_D levels and offspring bone parameters. Vitamin levels in cord blood were not associated with offspring BMD or TBS.

**Conclusions:**

This is the first study to show an association between maternal vitamin A status and offspring peak bone mass. Our findings may imply increase future risk for osteoporotic fracture in offspring of mothers with suboptimal vitamin A level. No associations were observed between 25(OH)D and 1,25(OH)_2_D and offspring BMD.

## Introduction

Increasing evidence suggests that the *in-utero* environment plays an important role in the development of future osteoporosis [1, 2]. Vitamin A and D are both important for bone health, and antenatal levels of these vitamins may exert critical influence on this process. Globally, about 20 million pregnant women are considered to be vitamin A deficient [3, 4]. Maternal vitamin D deficiency during pregnancy is a worldwide epidemic with a reported prevalence from 18–84% [5–7].

In the diet, vitamin A is obtained as retinyl ester or β-carotene, which are metabolized to retinol in the intestine [8]. In the circulation, retinol is incorporated in chylomicrons or bound to retinol binding protein and transthyretin. The liver is the main storage site for vitamin A, but a substantial amount is also transported to other tissues by chylomicrons, bone being the second most important organ for clearance of chylomicron remnants [8, 9]. All-*trans* retinoic acid, the biologically active form, binds to retinoic acid receptors (RARs) which heterodimerize with retinoid X receptors (RXRs) [8, 10, 11]. Vitamin A has been shown to promote bone formation and to inhibit generation of osteoclast progenitors [11]. The effects are, however, dependent on dose, and both hypo- and hypervitaminosis A may be harmful for the skeleton. Studies in rats show that vitamin A is essential for growth and normal skeletal development in the fetus, whereas excess retinol may have negative skeletal effects [12]. An inverse association between maternal serum retinol in late pregnancy and offspring total bone mineral content (BMC) was reported in neonates within 2 weeks after birth, whereas β-carotene was positively associated [13]. The association between maternal serum retinol during pregnancy and offspring bone health in adulthood has not been addressed.

Serum 25-hydroxyvitamin D [25(OH)D] is a measure of vitamin D status, and a precursor of the active form 1,25-dihydroxyvitamin D_3_ [1,25(OH)_2_D] which binds to the vitamin D receptor (VDR). Like RAR, VDR is dependent on heterodimerizing with RXR [14]. The primary action of vitamin D is to facilitate intestinal calcium absorption [15]. Severe vitamin D deficiency leads to rickets in children and osteomalacia in adults [15]. During pregnancy, a 2–3 time rise in 1,25(OH)_2_D concentration occurs to optimize calcium absorption and mineralization of the fetal skeleton [16, 17].

Data on the association between maternal 25(OH)D concentrations during pregnancy and offspring bone health are conflicting. Zhu *et al*. reported a positive association between maternal serum vitamin D level and offspring peak bone mass [18]. This was supported by a study showing that maternal 25(OH)D associated positively to offspring BMC at 9 years of age [19]. In contrast, two larger studies found no relationship between maternal serum 25(OH)D and offspring bone health at 6 and 9 years [20, 21]. No studies have evaluated the relation between maternal 1,25(OH)_2_D concentrations and adult offspring bone health.

Antagonizing effects of vitamin A and D have been reported in several studies [11, 22]. High levels of vitamin D seem to protect against vitamin A toxicity, whereas high vitamin A levels reduce the adverse effects of hypervitaminosis D [23]. High intake of vitamin A and concomitant low intake of vitamin D may enhance bone fragility [23–25]. It has been postulated that these opposing effects may be attributed to that both RAR and VDR heterodimerize with the RXR receptor [11, 26]. Thus, high levels of retinol could attenuate binding of the heterodimerized receptors VDR RXR to responsive elements of DNA, thereby impairing the actions of vitamin D.

We aimed to examine the association between maternal serum levels of retinol, 25(OH)D, and 1,25(OH)_2_D during second and third trimester and offspring BMD and trabecular bone score (TBS) at the age of peak bone mass (26 years).

## Methods

### Study design and participants

Mother-child pairs were recruited from a Caucasian-population-based prospective cohort study in Trondheim, Norway [27]. Pregnant women were followed from gestational week 17. Their term-born infants were followed up from birth. Mother-child pairs were included based on the access to maternal serum samples and offspring dual x-ray absorptiometry (DXA) scans. At inclusion, offspring age was 26 years. Exclusion criteria were cerebral palsy and pregnancy. The study was approved by the Regional Committee for Medical and Health Research Ethics in Central Norway (No: 2013/636/REK Mid-Norway). We obtained informed written consent from all participants.

### Procedures

Information on maternal sociodemographics, anthropometrics, and lifestyle factors were retrieved from the cohort data collected from 1986–1988. Maternal serum samples were collected at gestational weeks 17, 33, and 37, and in cord blood at delivery and stored at -80°C for later analyses. Analyses of all-trans retinol, hereafter referred to as retinol, and 25(OH)D were performed at BEVITAL Laboratory in Bergen, Norway using isotope-dilution liquid chromatography-tandem mass spectrometry (LC-MS/MS). The reference range for serum retinol was 1.5–2.8 μmol/L. Analysis of 1,25(OH)_2_D was performed at the Hormone Laboratory, Oslo University Hospital by an enzyme immunoassay (IDS Nordic A/S immunodiagnosticsystems). The reference range was 39–193 pmol/l (CV% 13 at 82 pmol/l).

At the age of 26 years, the offspring completed a questionnaire addressing calcium (from milk) and vitamin D intake, smoking and physical activity. Blood samples were collected after overnight fast and stored at -80° C until analyses. Analysis of 25(OH)D was performed at St. Olavs Hospital, Trondheim University Hospital, Norway. Offspring retinol was analyzed at the same laboratory and with the same method as the maternal samples.

BMD at lumbar spine (L1-L4), femoral neck, total hip and whole body, and spine TBS were measured with DXA applying Hologic Discovery A S/N 83817c (Hologic Bedford, MA, software version 13.4.2). The BMD results are presented in mg/cm^2^ and Z-score.

### Outcomes

Main outcomes were offspring BMD and TBS at the age of 26 years.

### Statistical analyses

Continuous variables are presented as mean and standard deviation (SD) or median and interquartile range (IQR) dependent on the data distribution. The student’s t-test or nonparametric test were applied. Differences in frequencies of categorical variables were analyzed by the Pearson chi-square test. Multivariate linear regression was used to examine associations between maternal serum retinol, 25(OH)D and 1,25(OH)_2_D, and offspring bone parameters. Retinol, 25(OH)D, and 1,25(OH)_2_D were treated as continuous exposure variables. A composite exposure variable was generated for each maternal serum parameter by averaging the concentration across three gestational time-points (weeks 17, 33 and 37). Adjustments were made for maternal age, preconception BMI, smoking and education, and offspring birth weight. These potential confounders were chosen based on previous studies [18–20], and as they were assumed to be associated both with exposure and outcome. Additional adjustment for offspring covariates (sex, BMI, smoking, physical activity and retinol and 25(OH)D levels) did not affect the results and were therefore not included in the analyses. Statistical analyses were conducted using SPSS statistics version 22·0 (IBM, Chicago, IL).

## Results

Forty-one mother-child pairs were included. Maternal data are presented in Tables 1 and 2.

**Table 1:** Anthropometric and sociodemographic characteristics of the mothers.

Maternal characteristics (n = 41)		
Age at delivery, yrs	29.6	(4.3)
Height, m	1.66	(0.07)
Weight, kg—preconception	58	(52–64)
- at 17-week gestation	59	(54–66)
BMI pre-conception, kg/m^2^	21.3	(3.1)
Smoking habits		
At conception (no/yes)		
Non-smoker	11	(27)
Smoker	30	(73)
At 17 week gestation		
Non-smoker	18	(44)
< 10 cigarettes per day	7	(17)
10–20 cigarettes per day	14	(34)
Unknown	2	(5)
Educational level		
Completed middle school only	4	(9.8)
Completed middle school plus 1–2 yrs	13	(31.7)
Completed high school	8	(19.5)
Higher education, non-university	13	(31.7)
University degree	3	(7.3)

Values are given as mean (standard deviation) or median (interquartile range) or number (%).

**Table 2:** Mean retinol, 25(OH)D and 1,25(OH)_2_D levels in maternal serum at different gestational weeks and in cord blood (n = 41).

Gestational week	Serum levels	
**Retinol, μmol/L**		
wk 17	1.82	(0.34)
wk 33	1.63	(0.42)
wk 37	1.54	(0.35)
Average	1.66	(0.32)
Cord blood	0.87	(0.24)
**25(OH)D, nmol/L**		
wk 17	57.8	(25.4)
wk 33	58.1	(27.8)
wk 37	57.6	(28.6)
Average	59.0	(20.6)
Cord blood	31.4	(18.6)
**1,25(OH)**_**2**_**D, pmol/L**		
wk 17	226.2	(71.2)
wk 33	273.6	(90.4)
wk 37	261.0	(69.5)
Average	251.3	(62.4)
Cord blood	116.5	(37.7)

Values are given as mean (standard deviation). 25(OH)D = 25-hydroxyvitamin D, 1,25(OH)_2_D = 1,25-hydroxyvitamin D.

Mean age at delivery was 29.6 (4.3) years and mean pre-conception BMI 21.3 (3.1) kg/m^2^. Seventy-three % of the women smoked at conception, and 51% smoked during pregnancy. The average retinol concentration across the three gestational time-points (week 17, 33 and 37) was 1.66 (0.32) μmol/L. Retinol levels declined significantly during pregnancy 1.82 (0.34) at 17 wk vs 1.63 (0.42) μmol/L at 33 wk, (CI -0.37, -0.01), p = 0.04, and 1.54 (0.35) μmol/L at 37 wk, (CI -0.44, -0.12), p = 0.001. Retinol inadequacy (< 1.05 μmol/L) was observed in one mother at week 33 and in five (12%) at week 37, 16 (39%) had levels below the reference level at week 37. Average 25(OH)D level during pregnancy was 59.0 (20.6) nmol/L. The levels remained relatively stable from second to third trimester. At week 17, 47.2% of the mothers had vitamin D insufficiency (25(OH)D < 50 nmol/L). The corresponding numbers at week 33 and 37 were 48.6 and 47.5%, respectively. Vitamin D deficiency (25(OH)D < 30 nmol/L) was observed in 11.1, 16.2 and 17.5% at week 17, 33 and 37, respectively.

Average 1,25(OH)_2_D concentration was 251.3 (62.4) pmol/L. A non-significant rise occurred between the trimesters. In week 37, mean level of 1,25(OH)_2_D in those with 25(OH)D levels < 30, between 30–50, between 50–75, and > 75 nmol/L did not differ (209, 253, 263 and 255 pmol/L, respectively, p = 0.726).

Offspring (n = 41) data are given in Table 3.

**Table 3:** Anthropometric, lifestyle and densitometry characteristics of offspring at age 26 years.

Offspring characteristics (n = 41)		
Age, years	26.1	(0.6)
Male	25	(61)
Height, m	1.76	(0.12)
Weight, kg	78.1	(18.3)
BMI, kg/m^2^	23.9	(21.9–27.4)
Birth weight, g	3410	(542)
Calcium intake (from milk), mg/d	85.0	(52.5–184.0)
Serum 25(OH)D, nmol/L	49.0	(35.0–78.5)
<50 nmol/L	21	(51.2)
≥50 nmol/L	20	(48.8)
Serum all-trans retinol, μmol/L	2.02	(0.46)
Daily physical activity, min	28.9	(5.9–32.1)
Smoking status		
Never	19	(46)
Former	17	(42)
Current	4	(10)
Unknown	1	(2)
Dual energy x-ray absorptiometry		
Bone mineral density, mg/cm^2^		
Lumbar spine	1015	(112)
Femoral neck	881	(123)
Total hip	1002	(124)
Whole body	1120	(83)
Bone mineral density, Z-score		
Lumbar spine	-0.505	(1.02)
Femoral neck	-0.071	(0.98)
Total hip	0.066	(0.87)
Whole body	-0.420	(0.94)
Trabecular bone score	1.42	(0.10)

Values are given as mean (standard deviation) or median (inter quartile range) or number (%).

BMI = body mass index, 25(OH)D = 25-hydroxyvitamin D

Among the offspring, 16 had birth weight below the 10^th^ percentile, and 25 above the 10^th^ percentile. Mean birth weight was 3410 (542) g. The age at inclusion was 26.1 (0.6) years; 61% (n = 25) were males. Mean BMI was 25.2 (5.0) kg/m^2^. None had vitamin A insufficiency, three had levels above the upper reference level (>2.8 μmol/L). Vitamin D insufficiency (25(OH)D <50 nmol/L) was observed in 51% (n = 21). The data collected on vitamin D intake were incomplete and did not allow estimation of daily intake. The median calcium intake from milk was 85 (52.5–184.0) mg/day.

Mean BMD Z-scores were -0.505 (1.02), -0.071 (0.98), 0.066 (0.87), and -0.420 (0.94), at the lumbar spine, femoral neck, total hip and whole body, respectively. Spine TBS was 1.42 (0.10). BMD and TBS did not differ significantly between participants with birth weight below and above the 10^th^ percentile (spine BMD: 1100 (70) and 1130 (90) mg/cm^2^, respectively, p = 0.28; TBS 1.40 (0.11) vs 1.43 (0.10), respectively, p = 0.40). Offspring spine BMD was significantly higher in those with maternal retinol levels above 1.54 μmol/L (mean level in week 37) compared to those with levels below [1040 (120) vs 960 (80) mg/cm^2^, p = 0.023]

Five subjects reported allergy, and three of them used antihistamine drugs; one had asthma and used glucocorticoid inhalation. Three had depression, of whom two were treated with antidepressants (escitalopram and sertraline). One participant had hypothyroidism and was adequately treated with levothyroxine. One subject with a history of eating disorder had normal BMI and BMD at inclusion. Another subject successfully treated for lymphoma also displayed satisfactory BMD. Additionally, four reported to have migraine and one chronic pain. Nine were currently using hormonal contraceptives and six were former users.

### Associations between maternal vitamin A and D levels and offspring bone parameters

After adjustment for maternal confounders and birth weight, offspring spine BMD and Z-score were positively associated with average retinol level across three gestational time-points, and increased by 30.8 (CI 7.6, 54.0) mg/cm^2^ and 0.24 (CI 0.03, 0.46) SD, respectively per 0.2 μmol/L increment in maternal retinol (Table 4).

**Table 4:** Associations of maternal serum retinol, 25(OH)D and 1,25(OH)_2_D during second and third trimester and offspring bone parameters at age 26 years.

	Δ Bone mineral density (mg/cm^2^) (n = 41)				Δ Z-score (n = 41)				Δ Trabecular bone score (n = 41)			
	Crude		Adjusted		Crude		Adjusted		Crude		Adjusted	
**Lumbar spine**												
Retinol per 0.2 μmol/L	21.6	(-0.04, 43.6)	30.8	(7.6, 54.0) *	0.16	(-0.04, 0.36)	0.24	(0.03, 0.46) *	**0**.012	(-0.008, 0.032)	0.021	(-0.002, 0.044)
25(OH)D per 10 nmol/L	-12.1	(-29.3, 5.0)	-10.0	(-30.0, 10.0)	-0.11	(-0.2**6**, 0.05)	-0.10	(-0.26, 0.07)	**0**.003	(-0.013, 0.019)	0.003	(-0.014, 0.020)
1,25(OH)_2_D per 25 pmol/L	-3.1	(-17.7, 11.5)	-2.3	(-17.4, 12.8)	-0.03	(-0.18, 0.10)	-0.03	(-0.16, 0.11)	**0**.001	(-0.012, 0.014)	-0.000	(-0.002, 0.015)
**Femoral neck**												
Retinol per 0.2 μmol/L	15.4	(-9.2, 40.0)	18.4	(-11.6, 48.4)	0.08	(-0.12, 0.28)	0.10	(-0.15, 0.34)				
25(OH)D per 10 nmol/L	1.7	(-17.5, 20.9)	2.0	(-19.7, 23.7)	0.01	(-0.14, 0.17)	0.20	(-0.16, 0.18)				
1,25(OH)_2_D per 25 pmol/L	-0.7	(-14.7, 16.2)	0.6	(-17.5, 18,8)	0.01	(-0.12, 0.13)	0.00	(-0.14, 0.15)				
**Total hip**												
Retinol per 0.2 μmol/L	21.8	(-2.6, 46.4)	25.2	(-4.6, 55.2)	0.10	(-0.06, 0.28)	0.13	(-0.09, 0.35)				
25(OH)D per 10 nmol/L	-1.3	(-20.8, 18.2)	-1.2	(-23.4, 20.9)	-0.01	(-0.15, 0.12)	-0.01	(-0.17, 0.14)				
1,25(OH)_2_D per 25 pmol/L	0.7	(-15.3, 16.7)	-0.2	(-18.5, 18.3)	0.01	(-0.11, 0.12)	0.00	(-0.13, 0.13)				
**Whole body**												
Retinol per 0.2 μmol/L	13.4	(-3.0, 29.6)	18.6	(-0.8, 38.2)	0.08	(-0.10, 0.28)	0.14	(-0.09, 0.37)				
25(OH)D per 10 nmol/L	0.4	(-12.6, 13.3)	-0.1	(-14.7, 14.5)	0.00	(-0.14, 0.15)	-0.00	(-0.17, 0.16)				
1,25(OH)_2_D per 25 pmol/L	6.0	(-4.7, 16.9)	5.8	(-6.3, 17.8)	0.08	(-0.05, 0.20)	0.07	(-0.07, 0.21)				

Values represent unstandardized linear regression coefficients B (crude and adjusted) and reflect the differences and 95% confidence intervals between increase in maternal retinol, 25(OH)D = 25-hydroxyvitamin D, and 1,25(OH)_2_D = 1,25-hydroxyvitamin D concentrations and adult offspring bone parameters. The dependent variable was adjusted for the following maternal covariates: age at delivery, preconception body mass index, educational level and smoking during pregnancy, and for offspring birth weight.

*p <0.05.

A positive, but non-significant (p = 0.08) association was also seen between maternal retinol levels and offspring TBS after adjustment (Table 4). Maternal retinol concentration at week 17 was positively associated with spine and whole-body BMD (S1 Table). Positive associations were also observed between retinol concentration at week 37 and offspring spine and total hip BMD (S3 Table). No associations were observed between average maternal 25(OH)D and 1,25(OH)_2_D levels and offspring BMD or TBS (Table 4). Retinol in cord blood was not associated with offspring BMD or TBS (spine, p = 0.77; femoral neck, p = 0.23; total hip, p = 0.31; whole body, p = 0.26; TBS, p = 0.47). Similarly, no associations were seen between 25(OH)D and 1,25(OH)_2_D levels in cord blood and offspring BMD or TBS (25(OH)D: BMD spine, p = 0.36; femoral neck, p = 0.99; total hip, p = 0.76; whole body, p = 0.88; TBS, p = 0.79. 1,25(OH)_2_D: spine, p = 0.52; femoral neck, p = 0.54; total hip, p = 0.61; whole body, p = 0.95; TBS, p = 0.57).

## Discussion

This is the first study to demonstrate a relationship between vitamin A status in pregnant women and offspring peak bone mass. After adjustment for maternal confounders and birth weight, we observed a significant positive association between average maternal retinol levels during second and third trimester and offspring spine BMD at 26 years of age. Offspring spine BMD Z-score increased by 0.24 SD per 0.2 μmol/L increment in maternal retinol. This is of clinical significance, as a BMD increase of 1 SD translates to a 2–3 times fracture reduction [28]. Retinol concentration at week 17 was also associated with offspring spine and whole body BMD and at week 37 with spine and total hip BMD. No associations were found between maternal 25(OH)D and 1,25(OH)_2_D levels and offspring bone parameters.

TBS is a measure of microarchitecture and bone quality, which has been shown to predict occurrence of major fractures independent of BMD [29]. We observed a positive association between average maternal retinol concentration and offspring TBS, albeit, not significant (p = 0.08). Impairment of bone quality in addition to lower peak bone mass in offspring of mothers with insufficient vitamin A status may enhance future fracture risk. In contrast to a study by Hyde *et al*.[30], no relation between maternal 25(OH)D concentration and TBS was found.

We show that low maternal vitamin A status may affect bone adversely and influence achievement of an optimal peak bone mass. This is of concern, as peak bone mass is regarded as the most important determinant of future fracture risk [31]. Our findings comply with studies in rodents demonstrating that retinol is mandatory for normal development of fetal bone [12]. Retinol plays a crucial role at an early stage in embryogenesis in patterning the entire axial skeleton and for development of other skeletal structures at a later stage [32]. The second trimester is a critical period for long bone growth [1], where vitamin A is an important actor, both deficiency and excess vitamin A causing inhibition of longitudinal growth [33]. In line with this, we observed a significant association between maternal retinol levels in week 17 and offspring BMD. Vitamin A deficiency in late embryogenic stage has been shown to affect development of the axial and appendicular skeleton in rats [12]. Accordingly, the significant association seen in week 37 in the present study, may indicate a role for vitamin A in skeletal development during the third trimester. As reviewed by Delgado-Calle et al., epidemiological and experimental studies suggest that epigenetic mechanisms influence skeletal development and the risk of osteoporosis [34]. Vitamin A is a potent epigenetic modulator that may produce long-term effects on the phenotype [35]. So far, there are no studies addressing epigenetic effects of vitamin A on the skeleton. Vitamin A is also involved in post-natal maintenance of bone [36]. *In vitro* studies suggest that retinol stimulates osteoblast differentiation and transition of osteoblasts to osteocytes and inhibits osteoclastogenesis, thus promoting bone formation [36–38].

Maternal vitamin D inadequacy may impair mineralization and thus affect the fetal skeleton directly [16, 17]. Moreover, skeletal effects may be attributed to epigenetic mechanisms, as vitamin D interacts with the epigenome at multiple levels [39, 40]. In contrast to Zhu *et al*. [18], we did not observe any association between maternal 25(OH)D and offspring BMD. There are no reports on the relation between maternal 1,25(OH)_2_D level and offspring BMD. In the present study, no association was found. In line with previous studies, maternal 1,25(OH)_2_D levels increased from second to third trimester. This rise occurs to facilitate calcium absorption and ensure mineralization of the fetal skeleton [16, 17]. We found that circulating 1,25(OH)_2_D was similar across different levels of 25(OH)D, illustrating that compensatory mechanisms may uphold serum 1,25(OH)_2_D levels in a state of maternal hypovitaminosis D [41]. Since vitamin D exerts its actions through 1,25(OH)_2_D, this could explain the lack of association between maternal 25(OH)D and offspring BMD. In a recent study including 855 pregnant women, we observed that 1,25(OH)_2_D level was significantly lower in women with 25(OH)D level < 30 nmol/L compared to those with level above 75 nmol/L [7]. Hollis *et al*. suggested that 25(OH)D levels of 100 nmol/L are necessary to obtain maximal 1,25(OH)_2_D concentrations during pregnancy [17]. The fact that none of our participants reached this level combined with the small sample size, may have attenuated the possibility to show associations.

In line with previous studies [13, 42], we observed a modest decline in retinol levels throughout pregnancy, which may be attributed to hemodilution, and depletion of maternal stores due to fetal demands. The fact that the majority of women were smokers could contribute to the inferior vitamin A status, as smoking seems to attenuate serum levels of beta-carotene [43]. Unfortunately, we do not have data on alterations in smoking status during pregnancy.

The optimal retinol concentration during pregnancy is not settled. Concentrations below 1.05 μmol/L are regarded as inadequate in the non-pregnant state. According to this criterion, five (12.5%) women displayed inadequacy in last trimester. In a meta-analysis addressing vitamin A and fracture risk, retinol levels between 1.99 and 2.31 μmol/L were suggested to be optimal [44]. A U-shaped relationship between serum retinol levels and hip fracture risk was observed, indicating that both high and low levels may be harmful [44]. Several studies have reported negative skeletal effects, however, mainly in those with retinol levels > 2.64 μmol/L[45]. In contrast, in a Norwegian study by Holvik *et al*.[46], hip fracture risk tended to be increased at low retinol concentrations, whereas high levels (up to 3 μmol/L) showed no adverse effect on hip fracture risk. In the present study, most of the women had retinol levels below 1.99 μmol/L, and about 40% had levels below the reference level. When stratifying offspring BMD according to mean maternal retinol level in week 37 (1.54 μmol/L), we observed a significantly higher spine BMD in offspring whose mothers had concentrations above this level.

Since vitamin A has to be obtained from the diet, it may be challenging to reach satisfactory levels [35]. Data from clinical trials indicate that a vegetarian diet alone is not sufficient to achieve an adequate vitamin A status [47]. A Cochrane review concluded that taking vitamin A supplements during pregnancy reduced the risk of anemia, infection and night blindness in the mother [47]. However, no reduction in deaths of mothers or newborns were seen [48]. The recommended intake during pregnancy differs between countries [49], and the adherence to these recommendations is low [50]. WHO advocates vitamin A supplementation only to pregnant women in regions with severe deficiency to prevent night blindness [3]. According to McCauley et al., the basal requirement during pregnancy is 370 μg/day and the recommended daily allowance 770 μg/dayhttps://www.ncbi.nlm.nih.gov/pubmed/26027509 [48]. It is not specified whether vitamin A supplements should be given as retinol ester or beta-carotene. In a study from rural Nepal, a positive effect on pulmonary function was observed in the offspring at the age of 9–13 years, in those whose mothers received supplements with preformed vitamin A during pregnancy. In children whose mothers were supplemented with beta-carotene, no beneficial effects on pulmonary function were observed [51, 52].

In the present study, almost 50% of the women exhibited vitamin D insufficiency at both second and third trimester. The occurrence of deficiency increased from 11.1 to 17.5% from week 17 to week 37. This is in accordance with previous studies showing a high prevalence of hypovitaminosis D during pregnancy [16, 17]. Infants whose mothers are vitamin D deficient may be born with hypocalcemia and some develop rickets and craniotabes [53]. The prenatal recommendations vary between countries, and the adherence seems to be poor [54].

Interference between vitamin A and D at receptor level could also affect fetal bone negatively. High levels of retinol combined with vitamin D deficiency could hinder binding of VDR to the heterodimer RAR-RXR and thus block the effects of vitamin D [55]. In the present study, none of the mothers with vitamin D deficiency had hypervitaminosis A, thus interaction between these vitamins is not likely to explain the association between maternal levels and offspring bone health.

The main limitation to the study is the small sample size, which makes it vulnerable to type 2 error, and to a lesser extent to type 1 error. The offspring comprised individuals born at term with low or normal birth weight. At the age of 26 years, they did, however, not differ significantly in BMI, BMD and TBS. We had access to maternal data that allowed adjustment for several confounding factors. However, information on potential confounding factors such as parity and dietary intake of vitamin A and D were lacking. Moreover, we did not have data on vitamin A and D intake in the offspring, and the calcium intake was underestimated as only milk consumption was recorded. The study participants were white, and the findings may thus not be applicable to other ethnic groups.

The study has several notable strengths, including the long-term follow-up. This enabled the assessment of offspring bone health at the age of peak bone mass, thus reducing the influence of factors as growth rate and developmental differences. Application of TBS for assessment of bone quality gave additional information concerning fracture risk. In contrast to most previous studies, serum levels of vitamin A and D were analyzed at several time-points across the last two trimesters of pregnancy and in cord blood at delivery, thus allowing assessment of at which stage of fetal skeletal development these vitamins may have the highest impact. Vitamin A levels preconception and in first trimester could have added further insight.

## Conclusion

Maternal retinol concentration during mid and late pregnancy was positively associated with offspring peak bone mass and bone quality. This may imply increase future fracture risk in offspring of mothers with inadequate vitamin A status. Our study contributes to novel knowledge on developmental origins of osteoporosis. Given the high prevalence of hypovitaminosis A worldwide, there is a need for increased attention to ensure sufficient intake during pregnancy. No associations between maternal 25(OH)D and 1,25(OH)_2_D concentrations and offspring bone health were shown. Studies with larger study populations are warranted to confirm our data.

## Supporting information

S1 Table Associations of maternal serum retinol, 25(OH)D and 1,25(OH)_2_D during gestational week 17 and offspring bone parameters at Age 26 years. (DOCX)

S2 Table Associations of maternal serum retinol, 25(OH)D and 1,25(OH)_2_D during gestational week 33 and offspring bone parameters at age 26 years. (DOCX)

S3 Table Associations of maternal serum retinol, 25(OH)D and 1,25(OH)_2_D during gestational week 37 and offspring bone parameters at age 26 years. (DOCX)

S1 File Background before mini interview. (PDF)

S2 File Physical health questionnaire. (PDF)

S3 File Pain and sleep questionnaire. (PDF)

S4 File Diet and eating habits questionnaire. (PDF)

S5 File Physical activity questionnaire. (PDF)

S6 File Questionnaire for women. (PDF)

S7 File Questionnaire for men. (PDF)

S8 File Norwegian Bakgrunn før mini interview. (PDF)

S9 File Norwegian Somatisk undersøkelse. (PDF)

S10 File Norwegian Smerter & søvn. (PDF)

S11 File Norwegian Kosthold og spisevaner. (PDF)

S12 File Norwegian Motorisk og fysisk aktivitet. (PDF)

S13 File Norwegian Spørsmål for kvinner. (PDF)

S14 File Norwegian Spørsmål for menn. (PDF)

## References

[1] Cooper C, Westlake S, Harvey N, Javaid K, Dennison E, Hanson M. Review: developmental origins of osteoporotic fracture. Osteoporosis international: a journal established as result of cooperation between the European Foundation for Osteoporosis and the National Osteoporosis Foundation of the USA. 2006;17(3):337–47. Epub 2005/12/07. 10.1007/s00198-005-2039-5 . 16331359

[2] Wood CL, Stenson C, Embleton N. The Developmental Origins of Osteoporosis. Current genomics. 2015;16(6):411–8. Epub 2015/12/. 10.2174/1389202916666150817202217 . 27018386 PMC4765528

[3] WHO. Global Prevalence of Vitamin A Deficiency in Populations at Risk 1995–2005. WHO Global Database on Vitamin A Deficiency. World Health Organization; Geneva, Switzerland 2009.

[4] Bastos Maia S, Rolland Souza AS, Costa Caminha MdF, Lins da Silva S, Callou Cruz RdSBL, Carvalho Dos Santos C, et al Vitamin A and Pregnancy: A Narrative Review. Nutrients. 2019;11(3):681 10.3390/nu11030681 . 30909386 PMC6470929

[5] Palacios C, Gonzalez L. Is vitamin D deficiency a major global public health problem? The Journal of steroid biochemistry and molecular biology. 2014;144 Pt A:138–45. Epub 2013/11/19. 10.1016/j.jsbmb.2013.11.003 24239505 PMC4018438

[6] Spiro A, Buttriss JL. Vitamin D: An overview of vitamin D status and intake in Europe. Nutrition bulletin. 2014;39(4):322–50. Epub 2015/01/31. 10.1111/nbu.12108 25635171 PMC4288313

[7] Gustafsson MK, Romundstad PR, Stafne SN, Helvik AS, Stunes AK, Morkved S, et al Alterations in the vitamin D endocrine system during pregnancy: A longitudinal study of 855 healthy Norwegian women. PloS one. 2018;13(4):e0195041 Epub 2018/04/12. 10.1371/journal.pone.0195041 29641551 PMC5895009

[8] Blomhoff R, Blomhoff HK. Overview of retinoid metabolism and function. Journal of neurobiology. 2006;66(7):606–30. Epub 2006/05/12. 10.1002/neu.20242 . 16688755

[9] Niemeier A, Niedzielska D, Secer R, Schilling A, Merkel M, Enrich C, et al Uptake of postprandial lipoproteins into bone in vivo: impact on osteoblast function. Bone. 2008;43(2):230–7. Epub 2008/06/10. 10.1016/j.bone.2008.03.022 . 18538644

[10] Soprano DR, Qin P, Soprano KJ. Retinoic acid receptors and cancers. Annual review of nutrition. 2004;24:201–21. Epub 2004/06/11. 10.1146/annurev.nutr.24.012003.132407 . 15189119

[11] Conaway HH, Henning P, Lerner UH. Vitamin a metabolism, action, and role in skeletal homeostasis. Endocrine reviews. 2013;34(6):766–97. Epub 2013/05/31. 10.1210/er.2012-1071 . 23720297

[12] See AW, Kaiser ME, White JC, Clagett-Dame M. A nutritional model of late embryonic vitamin A deficiency produces defects in organogenesis at a high penetrance and reveals new roles for the vitamin in skeletal development. Dev Biol. 2008;316(2):171–90. Epub 2008/03/07. 10.1016/j.ydbio.2007.10.018 . 18321479

[13] Handel MN, Moon RJ, Titcombe P, Abrahamsen B, Heitmann BL, Calder PC, et al Maternal serum retinol and beta-carotene concentrations and neonatal bone mineralization: results from the Southampton Women's Survey cohort. The American journal of clinical nutrition. 2016;104(4):1183–8. Epub 2016/09/16. 10.3945/ajcn.116.130146 27629051 PMC5039809

[14] Zhang J, Chalmers MJ, Stayrook KR, Burris LL, Wang Y, Busby SA, et al DNA binding alters coactivator interaction surfaces of the intact VDR-RXR complex. Nature structural & molecular biology. 2011;18(5):556–63. Epub 2011/04/12. 10.1038/nsmb.2046 21478866 PMC3087838

[15] Elder CJ, Bishop NJ. Rickets. The Lancet. 2014;383(9929):1665–76. 10.1016/S0140-6736(13)61650-5 24412049

[16] Kovacs CS. Maternal Mineral and Bone Metabolism During Pregnancy, Lactation, and Post-Weaning Recovery. Physiol Rev. 2016;96(2):449–547. Epub 2016/02/19. 10.1152/physrev.00027.2015 . 26887676

[17] Hollis BW, Johnson D, Hulsey TC, Ebeling M, Wagner CL. Vitamin D supplementation during pregnancy: double-blind, randomized clinical trial of safety and effectiveness. Journal of bone and mineral research: the official journal of the American Society for Bone and Mineral Research. 2011;26(10):2341–57. Epub 2011/06/28. 10.1002/jbmr.463 21706518 PMC3183324

[18] Zhu K, Whitehouse AJ, Hart PH, Kusel M, Mountain J, Lye S, et al Maternal vitamin D status during pregnancy and bone mass in offspring at 20 years of age: a prospective cohort study. Journal of bone and mineral research: the official journal of the American Society for Bone and Mineral Research. 2014;29(5):1088–95. Epub 2013/11/06. 10.1002/jbmr.2138 . 24189972

[19] Javaid MK, Crozier SR, Harvey NC, Gale CR, Dennison EM, Boucher BJ, et al Maternal vitamin D status during pregnancy and childhood bone mass at age 9 years: a longitudinal study. Lancet. 2006;367(9504):36–43. Epub 2006/01/10. 10.1016/S0140-6736(06)67922-1 . 16399151

[20] Lawlor DA, Wills AK, Fraser A, Sayers A, Fraser WD, Tobias JH. Association of maternal vitamin D status during pregnancy with bone-mineral content in offspring: a prospective cohort study. Lancet. 2013;381(9884):2176–83. Epub 2013/03/23. 10.1016/S0140-6736(12)62203-X 23518316 PMC3691477

[21] Garcia AH, Erler NS, Jaddoe VW, Tiemeier H, van den Hooven EH, Franco OH, et al 25-hydroxyvitamin D concentrations during fetal life and bone health in children aged 6 years: a population-based prospective cohort study. The lancet Diabetes & endocrinology. 2017 Epub 2017/03/06. 10.1016/s2213-8587(17)30064-5. . 28259646

[22] Lim LS, Harnack LJ, Lazovich D, Folsom AR. Vitamin A intake and the risk of hip fracture in postmenopausal women: the Iowa Women's Health Study. Osteoporosis international: a journal established as result of cooperation between the European Foundation for Osteoporosis and the National Osteoporosis Foundation of the USA. 2004;15(7):552–9. Epub 2004/02/05. 10.1007/s00198-003-1577-y 14760518 PMC2020807

[23] Caire-Juvera G, Ritenbaugh C, Wactawski-Wende J, Snetselaar LG, Chen Z. Vitamin A and retinol intakes and the risk of fractures among participants of the Women's Health Initiative Observational Study. The American journal of clinical nutrition. 2009;89(1):323–30. Epub 2008/12/06. 10.3945/ajcn.2008.26451 19056568 PMC2715292

[24] Mata-Granados JM, Cuenca-Acevedo R, Luque de Castro MD, Sosa M, Quesada-Gomez JM. Vitamin D deficiency and high serum levels of vitamin A increase the risk of osteoporosis evaluated by Quantitative Ultrasound Measurements (QUS) in postmenopausal Spanish women. Clinical biochemistry. 2010;43(13–14):1064–8. Epub 2010/07/06. 10.1016/j.clinbiochem.2010.06.001 . 20599880

[25] Mata-Granados JM, Cuenca-Acevedo JR, Luque de Castro MD, Holick MF, Quesada-Gomez JM. Vitamin D insufficiency together with high serum levels of vitamin A increases the risk for osteoporosis in postmenopausal women. Archives of osteoporosis. 2013;8:124 Epub 2013/02/19. 10.1007/s11657-013-0124-5 . 23417776

[26] Tata JR. Signalling through nuclear receptors. Nature reviews Molecular cell biology. 2002;3(9):702–10. Epub 2002/09/05. 10.1038/nrm914 . 12209130

[27] Bakketeig LS, Jacobsen GW, Hoffman HJ, Lindmark G, Bergsjo P, Molne K, et al Pre-pregnancy risk factors for small-for-gestational age births among parous women in Scandinavia. Acta obstetrica et gynecologica Scandinavica. 1993;72:273–9. 10.3109/00016349309068037 8389514

[28] Kanis JA, McCloskey EV, Johansson H, Oden A, Strom O, Borgstrom F. Development and use of FRAX in osteoporosis. Osteoporosis international: a journal established as result of cooperation between the European Foundation for Osteoporosis and the National Osteoporosis Foundation of the USA. 2010;21 Suppl 2:S407–13. Epub 2010/05/22. 10.1007/s00198-010-1253-y . 20464374

[29] Hans D, Goertzen AL, Krieg MA, Leslie WD. Bone microarchitecture assessed by TBS predicts osteoporotic fractures independent of bone density: the Manitoba study. Journal of bone and mineral research: the official journal of the American Society for Bone and Mineral Research. 2011;26(11):2762–9. Epub 2011/09/03. 10.1002/jbmr.499 . 21887701

[30] Hyde NK, Brennan-Olsen SL, Wark JD, Hosking SM, Holloway KL, Pasco JA. Maternal vitamin D and offspring trabecular bone score. Osteoporosis international: a journal established as result of cooperation between the European Foundation for Osteoporosis and the National Osteoporosis Foundation of the USA. 2017 Epub 2017/09/05. 10.1007/s00198-017-4208-8 . 28868588

[31] Weaver CM, Gordon CM, Janz KF, Kalkwarf HJ, Lappe JM, Lewis R, et al The National Osteoporosis Foundation's position statement on peak bone mass development and lifestyle factors: a systematic review and implementation recommendations. Osteoporosis international: a journal established as result of cooperation between the European Foundation for Osteoporosis and the National Osteoporosis Foundation of the USA. 2016;27(4):1281–386. Epub 2016/02/10. 10.1007/s00198-015-3440-3 26856587 PMC4791473

[32] Kaiser ME, Merrill RA, Stein AC, Breburda E, Clagett-Dame M. Vitamin A deficiency in the late gastrula stage rat embryo results in a one to two vertebral anteriorization that extends throughout the axial skeleton. Dev Biol. 2003;257(1):14–29. Epub 2003/04/25. 10.1016/s0012-1606(03)00044-7 . 12710954

[33] De Luca F, Uyeda JA, Mericq V, Mancilla EE, Yanovski JA, Barnes KM, et al Retinoic acid is a potent regulator of growth plate chondrogenesis. Endocrinology. 2000;141(1):346–53. Epub 1999/12/30. 10.1210/endo.141.1.7283 . 10614657

[34] Delgado-Calle J, Garmilla P, Riancho JA. Do epigenetic marks govern bone mass and homeostasis? Current genomics. 2012;13(3):252–63. Epub 2012/11/02. 10.2174/138920212800543129 23115526 PMC3382279

[35] Bar-El Dadon S, Reifen R. Vitamin A and the epigenome. Critical reviews in food science and nutrition. 2017;57(11):2404–11. Epub 2015/11/14. 10.1080/10408398.2015.1060940 . 26565606

[36] Green AC, Martin TJ, Purton LE. The role of vitamin A and retinoic acid receptor signaling in post-natal maintenance of bone. J Steroid Biochem Mol Biol. 2016;155(Pt A):135–46. Epub 2015/10/06. 10.1016/j.jsbmb.2015.09.036 . 26435449

[37] Mattinzoli D, Messa P, Corbelli A, Ikehata M, Zennaro C, Armelloni S, et al A novel model of in vitro osteocytogenesis induced by retinoic acid treatment. European cells & materials. 2012;24:403–25. Epub 2012/11/20. . 23160992 10.22203/ecm.v024a29

[38] Green AC, Poulton IJ, Vrahnas C, Hausler KD, Walkley CR, Wu JY, et al RARgamma is a negative regulator of osteoclastogenesis. The Journal of steroid biochemistry and molecular biology. 2015;150:46–53. Epub 2015/03/25. 10.1016/j.jsbmb.2015.03.005 . 25800721

[39] Fetahu IS, Hobaus J, Kallay E. Vitamin D and the epigenome. Front Physiol. 2014;5:164 Epub 2014/05/09. 10.3389/fphys.2014.00164 24808866 PMC4010791

[40] Harvey NC, Sheppard A, Godfrey KM, McLean C, Garratt E, Ntani G, et al Childhood bone mineral content is associated with methylation status of the RXRA promoter at birth. Journal of bone and mineral research: the official journal of the American Society for Bone and Mineral Research. 2014;29(3):600–7. Epub 2013/08/03. 10.1002/jbmr.2056 23907847 PMC3836689

[41] Moon RJ, Harvey NC, Cooper C. ENDOCRINOLOGY IN PREGNANCY: Influence of maternal vitamin D status on obstetric outcomes and the fetal skeleton. European journal of endocrinology. 2015;173(2):R69–83. Epub 2015/04/12. 10.1530/EJE-14-0826 25862787 PMC4968635

[42] Cruz S, Matos A, da Cruz SP, Pereira S, Saboya C, Ramalho A. Relationship between the Nutritional Status of Vitamin A per Trimester of Pregnancy with Maternal Anthropometry and Anemia after Roux-en-Y Gastric Bypass. Nutrients. 2017;9(9). Epub 2017/09/09. 10.3390/nu9090989 28885564 PMC5622749

[43] Faure H, Preziosi P, Roussel AM, Bertrais S, Galan P, Hercberg S, et al Factors influencing blood concentration of retinol, α-tocopherol, vitamin C, and β-carotene in the French participants of the SU.VI.MAX trial. European Journal of Clinical Nutrition. 2006;60(6):706–17. 10.1038/sj.ejcn.1602372 16391586

[44] Wu AM, Huang CQ, Lin ZK, Tian NF, Ni WF, Wang XY, et al The relationship between vitamin A and risk of fracture: meta-analysis of prospective studies. Journal of bone and mineral research: the official journal of the American Society for Bone and Mineral Research. 2014;29(9):2032–9. Epub 2014/04/05. 10.1002/jbmr.2237 . 24700407

[45] Chen GD, Zhu YY, Cao Y, Liu J, Shi WQ, Liu ZM, et al Association of dietary consumption and serum levels of vitamin A and beta-carotene with bone mineral density in Chinese adults. Bone. 2015;79:110–5. Epub 2015/06/02. 10.1016/j.bone.2015.05.028 . 26027509

[46] Holvik K, Ahmed LA, Forsmo S, Gjesdal CG, Grimnes G, Samuelsen SO, et al No increase in risk of hip fracture at high serum retinol concentrations in community-dwelling older Norwegians: the Norwegian Epidemiologic Osteoporosis Studies. The American journal of clinical nutrition. 2015;102(5):1289–96. Epub 2015/09/18. 10.3945/ajcn.115.110528 . 26377161

[47] Sommer A, Davidson FR. Assessment and control of vitamin A deficiency: the Annecy Accords. The Journal of nutrition. 2002;132(9 Suppl):2845s–50s. Epub 2002/09/11. 10.1093/jn/132.9.2845S . 12221259

[48] McCauley ME, van den Broek N, Dou L, Othman M. Vitamin A supplementation during pregnancy for maternal and newborn outcomes. Cochrane Database Syst Rev. 2015;(10):Cd008666 Epub 2015/10/28. 10.1002/14651858.CD008666.pub3 . 26503498 PMC7173731

[49] Haugen M, Brantsaeter AL, Alexander J, Meltzer HM. Dietary supplements contribute substantially to the total nutrient intake in pregnant Norwegian women. Annals of nutrition & metabolism. 2008;52(4):272–80. Epub 2008/07/23. 10.1159/000146274 18645244 PMC2813797

[50] Yakoob MY, Lo CW. Nutrition (Micronutrients) in Child Growth and Development: A Systematic Review on Current Evidence, Recommendations and Opportunities for Further Research. J Dev Behav Pediatr. 2017;38(8):665–79. Epub 2017/07/27. 10.1097/DBP.0000000000000482 . 28746059

[51] Checkley W, West KP, Wise RA, Baldwin MR, Wu L, LeClerq SC, et al Maternal Vitamin A Supplementation and Lung Function in Offspring. New England Journal of Medicine. 2010;362(19):1784–94. 10.1056/NEJMoa0907441 . 20463338

[52] Bastos Maia S, Costa Caminha MF, Lins da Silva S, Rolland Souza AS, Carvalho Dos Santos C, Batista Filho M. The Prevalence of Vitamin A Deficiency and Associated Factors in Pregnant Women Receiving Prenatal Care at a Reference Maternity Hospital in Northeastern Brazil. Nutrients. 2018;10(9). Epub 2018/09/13. 10.3390/nu10091271 30205601 PMC6165532

[53] Soliman A, Salama H, Alomar S, Shatla E, Ellithy K, Bedair E. Clinical, biochemical, and radiological manifestations of vitamin D deficiency in newborns presented with hypocalcemia. Indian J Endocrinol Metab. 2013;17(4):697–703. Epub 2013/08/21. 10.4103/2230-8210.113764 23961489 PMC3743373

[54] Wirth JP, Petry N, Tanumihardjo SA, Rogers LM, McLean E, Greig A, et al Vitamin A Supplementation Programs and Country-Level Evidence of Vitamin A Deficiency. Nutrients. 2017;9(3). Epub 2017/03/02. 10.3390/nu9030190 28245571 PMC5372853

[55] Thompson PD, Remus LS, Hsieh JC, Jurutka PW, Whitfield GK, Galligan MA, et al Distinct retinoid X receptor activation function-2 residues mediate transactivation in homodimeric and vitamin D receptor heterodimeric contexts. Journal of molecular endocrinology. 2001;27(2):211–27. Epub 2001/09/21. . 11564604 10.1677/jme.0.0270211

