# Supplementary material for: Maternal serum retinol, 25(OH)D and 1,25(OH)2D concentrations during pregnancy and peak bone mass and trabecular bone score in adult offspring at 26-year follow-up
Source: PLoS One. 2019 Sep 26;14(9):e0222712. doi: 10.1371/journal.pone.0222712 (PMC6762137; doi:10.1371/journal.pone.0222712)
Supplement: S1 Table — (DOCX) [file pone.0222712.s001.docx]

**S1 Table: Associations of maternal serum retinol, 25(OH)D and 1,25(OH)_2_D during gestational week 17 and offspring bone parameters at age 26 years**

|  | **Δ Bone mineral density (mg/cm^2^) (n=41)** | | | | **Δ Z-score (n=41)** | | | | **Δ Trabecular bone score (n=41)** | | | | |
| --- | --- | --- | --- | --- | --- | --- | --- | --- | --- | --- | --- | --- | --- |
|  |  | |  | |  | |  | |  | |  | | |
|  | **Crude** | | **Adjusted** |  | **Crude** | | **Adjusted** |  | **Crude** | | **Adjusted** | |  |
| **Lumbar spine** |  |  |  |  |  |  |  |  |  |  |  |  | |
| Retinol per 0.2 µmol/L | 20.8 | (-1.2, 42.8) | 30.2 | (5.8, 54.6)* | 0.14 | (-0.06, 0.34) | 0.23 | (0.01, 0.46)* | **0.**002 | (-0.020, 0.022) | 0.012 | (-0.013, 0.037) | |
| 25(OH)D per 10 nmol/L | 0.2 | (-20.0, 20.0) | -2.0 | (-17.6, 13.6) | 0.02 | (-0.11, 0.16) | 0.01 | (-0.14, 0.15) | **0.**003 | (-0.011, 0.017) | 0.002 | (-0.012, 0.017) | |
| 1,25(OH)_2_D per 25 pmol/L | -2.1 | (-15.0, 10.9) | -1.6 | (-15.0, 11.8) | -0.01 | (-0.12, 0.11) | -0.00 | (-0.12, 0.12) | **0.**000 | (-0.011, 0.012) | 0.002 | (-0.011, 0.015) | |
| **Femoral neck** |  |  |  |  |  |  |  |  |  |  |  |  | |
| Retinol per 0.2 µmol/L | 11.4 | (-13.8, 36.4) | 17.8 | (-13.8, 49.4) | 0.02 | (-0.18, 0.22) | 0.07 | (-0.18, 0.33) |  |  |  |  | |
| 25(OH)D per 10 nmol/L | 4.5 | (-12.3, 21.3) | 3.8 | (-14.9, 22.4) | 0.05 | (-0.08, 0.18) | 0.05 | (-0.10, 0.19) |  |  |  |  | |
| 1,25(OH)_2_D per 25 pmol/L | 0.7 | (-13.3, 14.7) | 0.8 | (-15.3, 16.9) | 0.03 | (-0.10, 0.13) | 0.02 | (-0.11, 0.15) |  |  |  |  | |
| **Total hip** |  |  |  |  |  |  |  |  |  |  |  |  | |
| Retinol per 0.2 µmol/L | 22.2 | (-2.0, 46.8) | 30.3 | (-0.4, 61.0) | 0.10 | (-0.08, 0.28) | 0.15 | (-0.07, 0.38) |  |  |  |  | |
| 25(OH)D per 10 nmol/L | 4.9 | (-11.9, 21.7) | 5.4 | (-13.5, 24.3) | 0.06 | (-0.06, 0.17) | 0.06 | (-0.07, 0.20) |  |  |  |  | |
| 1,25(OH)_2_D per 25 pmol/L | 0.3 | (-14.3, 14.9) | -0.5 | (-16.9, 15.9) | 0.03 | (-0.08, 0.13) | 0.02 | (-0.10, 0.13) |  |  |  |  | |
| **Whole body** |  |  |  |  |  |  |  |  |  |  |  |  | |
| Retinol per 0.2 µmol/L | 16.4 | (1.0, 31.8)* | 26.8 | (7.6, 45.8)* | 0.10 | (-0.08, 0.28) | 0.21 | (-0.02, 0.44) |  |  |  |  | |
| 25(OH)D per 10 nmol/L | 4.8 | (-6.0, 15.5) | 4.2 | (-8.2, 16.7) | 0.09 | (-0.03, 0.20) | 0.09 | (-0.05, 0.23) |  |  |  |  | |
| 1,25(OH)_2_D per 25 pmol/L | 3.3 | (-6.5, 13.1) | 4.0 | (-6.7, 14.7) | 0.05 | (-0.05, 0.18) | 0.08 | (-0.04, 0.20) |  |  |  |  | |

Values represent unstandardized linear regression coefficients B (crude and adjusted) and reflect the differences and 95% confidence intervals between increase in maternal retinol, 25(OH)D=25-hydroxyvitamin D, and 1,25(OH)_2_D=1,25-hydroxyvitamin D concentrations and adult offspring bone parameters. Dependent variable was adjusted for the following maternal covariates: age at delivery, preconception body mass index, educational level and smoking during pregnancy, and for offspring birth weight. **p* <0.05
