# Supplementary material for: Maternal serum retinol, 25(OH)D and 1,25(OH)2D concentrations during pregnancy and peak bone mass and trabecular bone score in adult offspring at 26-year follow-up
Source: PLoS One. 2019 Sep 26;14(9):e0222712. doi: 10.1371/journal.pone.0222712 (PMC6762137; doi:10.1371/journal.pone.0222712)
Supplement: S2 Table — (DOCX) [file pone.0222712.s002.docx]

**S2 Table: Associations of maternal serum retinol, 25(OH)D and 1,25(OH)_2_D during gestational week 33 and offspring bone parameters at age 26 years**

|  | **Δ Bone mineral density (mg/cm^2^) (n=41)** | | | | **Δ Z-score (n=41)** | | | | | **Δ Trabecular bone score (n=41)** | | | | |
| --- | --- | --- | --- | --- | --- | --- | --- | --- | --- | --- | --- | --- | --- | --- |
|  |  | |  | |  | |  | | |  | |  | | |
|  | **Crude** | | **Adjusted** |  | **Crude** | | **Adjusted** | |  | **Crude** | | **Adjusted** | |  |
| **Lumbar spine** |  |  |  |  |  |  |  |  | |  |  |  |  | |
| Retinol per 0.2 µmol/L | 12.4 | (-5.6, 30.2) | 14.8 | (-5.6, 35.4) | 0.10 | (-0.06, 0.26) | 0.11 | (-0.08, 0.30) | | **0.**014 | (-0.002, 0.030) | 0.018 | (-0.001, 0.037) | |
| 25(OH)D per 10 nmol/L | -11.5 | (-24.8, 1.8) | -11.2 | (-25.5, 3.2) | -0·10 | (-0.22, 0.02) | -0.09 | (-0.23, 0.04) | | **0.**002 | (-0.010, 0.015) | 0.002 | (-0.012, 0.016) | |
| 1,25(OH)_2_D per 25 pmol/L | -7.0 | (-17.5, 3.5) | -5.2 | (-16.4, 5.9) | -0.08 | (-0.18, 0.03) | -0.06 | (-0.16, 0.05) | | **-0.**003 | (-0.013, 0.007) | -0.003 | (-0.014, 0.007) | |
| **Femoral neck** |  |  |  |  |  |  |  |  | |  |  |  |  | |
| Retinol per 0.2 µmol/L | 3.2 | (-16.4, 22.6) | 0.8 | (-26.2, 24.6) | 0.00 | (-0.16, 0.16) | -0.04 | (-0.24, 0.16) | |  |  |  |  | |
| 25(OH)D per 10 nmol/L | -4.8 | (-19.4, 9.8) | -5.9 | (-23.6, 11.9) | -0.03 | (-0.15, 0.09) | -0.03 | (-0.17, 0.11) | |  |  |  |  | |
| 1,25(OH)_2_D per 25 pmol/L | -3.7 | (-15.2, 7.8) | -3.7 | (-17.2, 9.7) | -0.03 | (-0.13, 0.05) | -0.03 | (-0.14, 0.07) | |  |  |  |  | |
| **Total hip** |  |  |  |  |  |  |  |  | |  |  |  |  | |
| Retinol per 0.2 µmol/L | 5.6 | (-14.6, 25.6) | 2.8 | (-23.0, 28.6) | 0.00 | (-0.14, 0.16) | -0.01 | (-0.20, 0.17) | |  |  |  |  | |
| 25(OH)D per 10 nmol/L | -8.8 | (-23.6, 6.1) | -11.1 | (-28.8, 6.6) | -0.06 | (-0.16, 0.05) | -0.07 | (-0.20, 0.06) | |  |  |  |  | |
| 1,25(OH)_2_D per 25 pmol/L | -3.2 | (-15.3, 8.8) | -3.5 | (-17.3, 10.2) | -0.03 | (-0.10, 0.03) | -0.03 | (-0.13, 0.07) | |  |  |  |  | |
| **Whole body** |  |  |  |  |  |  |  |  | |  |  |  |  | |
| Retinol per 0.2 µmol/L | 4.8 | (-8.8, 18.4) | 5.2 | (-11.8, 22.2) | 0.02 | (-0.14, 0.16) | 0.01 | (-0.18, 0.21) | |  |  |  |  | |
| 25(OH)D per 10 nmol/L | -3.6 | (-13.9, 6.6) | -4.9 | (-16.8, 7.0) | -0.03 | (-0.15, 0.09) | -0.04 | (-0.18, 0.10) | |  |  |  |  | |
| 1,25(OH)_2_D per 25 pmol/L | 0.7 | (-7.4, 8.9) | 1.2 | (-8.0, 10.3) | 0.00 | (-0.08, 0.10) | 0.01 | (-0.10, 0.11) | |  |  |  |  | |

Values represent unstandardized linear regression coefficients B (crude and adjusted) and reflect the differences and 95% confidence intervals between increase in maternal retinol, 25(OH)D=25-hydroxyvitamin D, and 1,25(OH)_2_D=1,25-hydroxyvitamin D concentrations and adult offspring bone parameters. Dependent variable was adjusted for the following maternal covariates: age at delivery, preconception body mass index, educational level and smoking during pregnancy, and for offspring birth weight. **p* <0.05
