# Supplementary material for: Maternal serum retinol, 25(OH)D and 1,25(OH)2D concentrations during pregnancy and peak bone mass and trabecular bone score in adult offspring at 26-year follow-up
Source: PLoS One. 2019 Sep 26;14(9):e0222712. doi: 10.1371/journal.pone.0222712 (PMC6762137; doi:10.1371/journal.pone.0222712)
Supplement: S3 Table — (DOCX) [file pone.0222712.s003.docx]

**S3 Table: Associations of maternal serum retinol, 25(OH)D and 1,25(OH)_2_D during gestational week 37 and offspring bone parameters at age 26 years**

|  | **Δ Bone mineral density (mg/cm^2^) (n=41)** | | | | **Δ Z-score (n=41)** | | | | | **Δ Trabecular bone score (n=41)** | | | | |
| --- | --- | --- | --- | --- | --- | --- | --- | --- | --- | --- | --- | --- | --- | --- |
|  |  | |  | |  | |  | | |  | |  | | |
|  | **Crude** | | **Adjusted** |  | **Crude** | | **Adjusted** | |  | **Crude** | | **Adjusted** | |  |
| **Lumbar spine** |  |  |  |  |  |  |  |  | |  |  |  |  | |
| Retinol per 0.2 µmol/L | 16.6 | (-3.6, 36.8) | 26.8 | (7.2, 46.6)* | 0.12 | (-0.06, 0.32) | 0.22 | (0.03, 0.40)* | | **0.**008 | (-0.104, 0.272) | 0.013 | (-0.007, 0.034) | |
| 25(OH)D per 10 nmol/L | -4.1 | (-17.0, 8.8) | -2,9 | (-15.6, 9.9) | -0.05 | (-0.16, 0.07) | -0.04 | (-0.15, 0.08) | | **0.**003 | (-0.009, 0.015) | 0.002 | (-0.010, 0.015) | |
| 1,25(OH)_2_D per 25 pmol/L | 1.8 | (-11.3, 15.0) | 1.4 | (-12.2, 15.0) | 0.0 | (-0.11, 0.12) | 0.0 | (-0.12, 0.12) | | **0.**001 | (-0.010, 0.013) | 0.001 | (-0.012, 0.014) | |
| **Femoral neck** |  |  |  |  |  |  |  |  | |  |  |  |  | |
| Retinol per 0.2 µmol/L | 20.0 | (-1.8, 41.8) | 24.6 | (-0.2, 49.4) | -0.14 | (-0.04, 0.30) | 0.17 | (-0.03, 0.37) | |  |  |  |  | |
| 25(OH)D per 10 nmol/L | 8.3 | (-5.5, 22.2) | 8.4 | (-6.6, 23.5) | -0.05 | (-0.06, 0.16) | 0.05 | (-0.07, 0.17) | |  |  |  |  | |
| 1,25(OH)_2_D per 25 pmol/L | 5.3 | (-8.5, 19.0) | 4.7 | (-11.4, 20.9) | 0.03 | (-0.08, 0.14) | 0.03 | (-0.10, 0.15) | |  |  |  |  | |
| **Total hip** |  |  |  |  |  |  |  |  | |  |  |  |  | |
| Retinol per 0.2 µmol/L | 25.0 | (2.8, 115.0)* | 29.0 | (4.4, 53.6)* | 0.14 | (-0.02, 0.30) | 0.18 | (-0.00, 0.35) | |  |  |  |  | |
| 25(OH)D per 10 nmol/L | 5.4 | (-8.8, 19.7) | 5.2 | (-10.3, 20.8) | 0.02 | (-0.08, 0.12) | 0.02 | (-0.09, 0.13) | |  |  |  |  | |
| 1,25(OH)_2_D per 25 pmol/L | 6.3 | (-7.9, 20.5) | 4.9 | (-11.5, 21.3) | 0.03 | (-0.08, 0.13) | 0.03 | (-0.10, 0.13) | |  |  |  |  | |
| **Whole body** |  |  |  |  |  |  |  |  | |  |  |  |  | |
| Retinol per 0.2 µmol/L | 10.0 | (-5.2, 25.0) | 13.8 | (-3.2, 30.8) | 0.38 | (-0.28, 1.24) | 0.12 | (-0.81, 0.31) | |  |  |  |  | |
| 25(OH)D per 10 nmol/L | 5.0 | (-4.4, 14.4) | 5.0 | (-5.2, 15.1) | 0.03 | (-0.08, 0.14) | 0.03 | (-0.09, 0.15) | |  |  |  |  | |
| 1,25(OH)_2_D per 25 pmol/L | 6.7 | (-2.9, 16.3) | 6.4 | (-4.2, 17.1) | 0.05 | (-0.05, 0.15) | 0.05 | (-0.08, 0.18) | |  |  |  |  | |

Values represent unstandardized linear regression coefficients B (crude and adjusted) and reflect the differences and 95% confidence intervals between increase in maternal retinol, 25(OH)D=25-hydroxyvitamin D, and 1,25(OH)_2_D=1,25-hydroxyvitamin D concentrations and adult offspring bone parameters. Dependent variable was adjusted for the following maternal covariates: age at delivery, preconception body mass index, educational level and smoking during pregnancy, and for offspring birth weight. **p* <0.05
