# Supplementary material for: Maternal serum retinol, 25(OH)D and 1,25(OH)2D concentrations during pregnancy and peak bone mass and trabecular bone score in adult offspring at 26-year follow-up
Source: PLoS One. 2019 Sep 26;14(9):e0222712. doi: 10.1371/journal.pone.0222712 (PMC6762137; doi:10.1371/journal.pone.0222712)
Supplement: S1 File — (PDF) [file pone.0222712.s004.pdf]

BACKGROUND INFORMATION (interview)

Sex: 0 ☐ **Female** 1 ☐ **Male** **Ethnicity** ________________________________

Whom do you presently live with? (select only one alternative)

1 ☐ Parents/guardian 2 ☐ Siblings 3 ☐ Remaining family

4 ☐ Alone at bed-sit 5 ☐ Alone in apartment 6 ☐ Shared housing

7 ☐ Partner/spouse 8 ☐ Residence with inspection 9 ☐ Other – describe: ______________

EV. supplemental information: ____

Marital status:

1 ☐ Married 2 ☐ Cohabitant 3 ☐ Divorced 4 ☐ Single

SIBLINGS: Sex and age

0 ☐ Female 1 ☐ Male ________ years 0 ☐ Female 1 ☐ Male ________ years

0 ☐ Female 1 ☐ Male ________ years 0 ☐ Female 1 ☐ Male ________ years

If more, enter: ____

EDUCATION, OCCUPATION AND ECONOMY

1 ☐ In education 2 ☐ Employed 3 ☐ Benefit recipient 4 ☐ Unemployed

EV. supplemental information: ____

COMPLETED education:

1 ☐ Primary school (1.-7. grade)
2 ☐ Lower secondary school (8.-10. grade)
3 ☐ Upper secondary school – level 1
4 ☐ Upper secondary school – level 2
5 ☐ Upper secondary school – general university/college admissions certification
6 ☐ Upper secondary school extension – general university/college admissions certificate
7 ☐ Training as 2-year apprentice with certificate
8 ☐ Non-degree granting college
9 ☐ Vocational school 1-2 years: ___________________
10 ☐ Bachelor: ______________________________
11 ☐ Master:______________________________
12 ☐ Professional study: ________________________
13 ☐ Doctorate (PhD): __________________________
14 ☐ Unknown/other

Any supplemental information: ______________________
____

Education completed at normed time?

0 ☐ No 1 ☐ Yes

Present occupation: ____

Your total income last year: 1 ☐ < 100.000 2 ☐ 100-199.000 3 ☐ 200-349.000
4 ☐ 350-549.000 5 ☐ 550-749.000 6 ☐ ≥ 750.000

How will you describe your economy:

1 ☐ Very bad 2 ☐ Bad 3 ☐ Average 4 ☐ Good 5 ☐ Very good

Are you completely dependent on economic support from your spouse: 0 ☐ No 1 ☐ Yes

Are you completely dependent on economic support from your parents/family: 0 ☐ Nei 1 ☐ Ja

SOMATIC AND PSYCHIATRIC CASE HISTORY

Who is your family doctor? ______________________________

Do you have any disease/affliction that requires regular follow-up or check-up? (somatic disease, e.g. asthma, diabetes, epilepsy etc.)

0 ☐ No 1 ☐ Yes Which: ______________________________

Do you get regular follow-up presently? 0 ☐ No 1 ☐ Yes Clarify for what: ______________________

With whom (name of doctor/others, or ward/hospital): ______________________________

Are you taking any medication (including asthma inhalation) *presently?* 0 ☐ No 1 ☐ Yes

Drug name: ______________________ Dosage: ______ Daily (check): ____ As needed (check) : _____

Drug name: ______________________ Dosage: ______ Daily (check): ____ As needed (check) : _____

EV. earlier: 0 ☐ No 1 ☐ Yes

Drug name: ______________________ Dosage: ______ From ___.___.___ To ___.___.___

Drug name: ______________________ Dosage: ______ From ___.___.___ To ___.___.___

Ever admitted to hospital? 0 ☐ No 1 ☐ yes For what: ______________________

Severe physical injuries? 0 ☐ No 1 ☐ Yes What kind of:______________________

Head injury? (Specify whether was unconscious): 0 ☐ No 1 ☐ Yes What kind of: ______________

Other somatic problems presently/before: 0 ☐ No 1 ☐ Yes What: ______________

Hearing

Reduced hearing? 0 ☐ No 1 ☐ Yes If **yes**, the deviation? ______________________

Vision

Reduced vision? 0 ☐ No 1 ☐ Yes

Uses glasses? 0 ☐ oi 1 ☐ Yes – for reading or pc 2 ☐ Yes – regular use

If **yes**, why? (cause of sisual reduction) ______________________________

Learning disabilities

Do you have learning disabilities? 0 ☐ No 1 ☐ Yes

If **yes**, which?

1 ☐ Reading/writing disability

2 ☐ Math disability

3 ☐ Other, describe ______________________

Which investigation has been performed?

____

What kind of help did you get? ______________________________

Motor disabilities

Do you have motor difficulties (feel awkward, coordination difficulties etc.)? 0 ☐ No 1 ☐ Yes

If **yes**, describe: ______________________________

MENTAL DISTRESS

Presently: 0 ☐ No 1 ☐ Yes

____

____

____

Previously: 0 ☐ No 1 ☐ Yes

____

____

____

Do you get help for mental distress now? 0 ☐ No 1 ☐ Yes

Have you received help for mental distress previously? 0 ☐ No 1 ☐ Yes

If **yes**, from whom?

1 ☐ Psychologist 2 ☐ Psychiatrist 3 ☐ General practitioner 4 ☐ Health nurse

5 ☐ Nurse 6 ☐ Educational psychological service

Outpatient treatment	0 ☐ No	1 ☐ Yes	Age: ___ years
Day patient/ambulant team	0 ☐ No	1 ☐ Yes	Age: ___ years
Hospital admittance	0 ☐ No	1 ☐ Yes	Age: ___ years

Number of admissions to psychiatric hospital ____ EV. name of hospital:____________________________________

Medicine List (Psychopharmaca) presently:

1	Antipsychotics ________________<br>______________________________	0 ☐ No	1 ☐ Yes	4	Stimulantia _________________<br>___________________________	0 ☐ No	1 ☐ Yes
2	Antidepressiva ________________<br>______________________________	0 ☐ No	1 ☐ Yes	5	Mood stabilizers _____________<br>___________________________	0 ☐ No	1 ☐ Yes
3	Sedatives _____________________<br>______________________________	0 ☐ No	1 ☐ Yes	6	Other ______________________<br>___________________________	0 ☐ No	1 ☐ Yes

Comment after the introduction: ____
