# Supplementary material for: Maternal serum retinol, 25(OH)D and 1,25(OH)2D concentrations during pregnancy and peak bone mass and trabecular bone score in adult offspring at 26-year follow-up
Source: PLoS One. 2019 Sep 26;14(9):e0222712. doi: 10.1371/journal.pone.0222712 (PMC6762137; doi:10.1371/journal.pone.0222712)
Supplement: S2 File — (PDF) [file pone.0222712.s005.pdf]

A LITTLE MORE ABOUT YOUR PHYSICAL HEALTH (interview)

1. Do you have (had) high blood pressure? Previously: ☐ No ☐ Yes
Presently: ☐ No ☐ Yes

2. Do you get check-up from specialist for high blood pressure PRESENTLY? ☐ No ☐ Yes

3. Do you take blood pressure medications now? ☐ No ☐ Yes

---

4. Do you have (had) heart problems? Previously: ☐ No ☐ Yes
Presently: ☐ No ☐ Yes

5. Do you get check-up from specialist for heart problems PRESENTLY? ☐ No ☐ Yes

6. Do you take medication for heart problems now? ☐ No ☐ Yes

---

7. Do you have (had) lung problems?
a) Asthma: Previously: ☐ No ☐ Yes Presently: ☐ No ☐ Yes
b) Bronchitis: Previously: ☐ No ☐ Yes Presently: ☐ No ☐ Yes
c) Pneumonia: Previously: ☐ No ☐ Yes Presently: ☐ No ☐ Yes

8. Have you been admitted to the hospital for lung problems? ☐ No ☐ Yes

9. Do you get check-up from specialist for lung problems PRESENTLY? ☐ No ☐ Yes

10. Do you take medication for lung problems presently? ☐ No ☐ Yes

11. Smoked/smoke?
Previously: ☐ No ☐ Yes Number of sigarettes daily: ____ For how long (years): _____
Presently: ☐ No ☐ Yes Number of sigarettes daily: ____ For how long (years): _____

12. Snuff?
Previously: ☐ No ☐ Yes Number of pinches daily: ____ For how long (years): _____
Presently: ☐ No ☐ Yes Number of pinches daily: ____ For how long (years): _____

---

13. Do you have diabetes? ☐ No ☐ Yes ☐ Diabetes 1 ☐ Diabetes 2

14. If Yes, when did you get diabetes?_____ years of age

15. Are you taking medication for diabetes? ☐ No ☐ Yes, tablets ☐ Yes, insulin (inject)

---

16. Have you had a bone fracture? ☐ No ☐ Yes At age:____ years (if several, enter all)

17. If Yes, location (if several, enter all)): ____

18. If Yes, how did it happen? (describe): ______________________________________
Low energy fracture ☐ Traumatic fracture ☐

---

19. Do you have (had) epilepsy? Previously: ☐ No ☐ Yes Presently: ☐ No ☐ Yes

20. Do you get check-up from specialist for epilepsy PRESENTLY? ☐ No ☐ Yes

21. Do you take medication for lung problems presently? ☐ No ☐ Yes

---

22. Do you have cerebral palsy? ☐ No ☐ Yes

23. Other diseases (e.g. joint pain/rheumatic affliction, other): ______________________

EXAMINATION: Examiner: _____________ Date: ___________

Blood pressuire sitting, right arm after 5 min rest 3 times with 2 min interval:

	Systolic BP	Diastolic BP	MAP	HR
1. measurement				
2. measurement				
3. measurement				

Head circumference: _____ cm (one decimal)

Upper arm length: Right _____ cm (one decimal) Left _____ cm (one decimal)

Upper arm circumf: Right _____ cm (one decimal) Left _____ cm (one decimal)

Waist circumf. across navel: _____ cm (one decimal)

Waist circumf. between iliac crest and lower rib: _____ cm (one decimal)

Hip circumference: _____ cm (one decimal)

Skinfolds:

1. Left triceps skinfold
   1. measurement: _____ mm (one decimal)
   2. measurement: _____ mm (one decimal)
2. Left subscapular skinfold
   1. measurement: _____ mm (one decimal)
   2. measurement: _____ mm (one decimal)
