# Supplementary material for: Maternal serum retinol, 25(OH)D and 1,25(OH)2D concentrations during pregnancy and peak bone mass and trabecular bone score in adult offspring at 26-year follow-up
Source: PLoS One. 2019 Sep 26;14(9):e0222712. doi: 10.1371/journal.pone.0222712 (PMC6762137; doi:10.1371/journal.pone.0222712)
Supplement: S3 File — (PDF) [file pone.0222712.s006.pdf]

PAIN

1. Do you have bodily pain presently, that has lasted for <u>more than 6 months?</u>?

Yes	No
☐	☐

2. How severe bodily pain have you experienced during <u>the last 4 weeks</u>?

None	Very weak	Weak	Moderate	Severe	Very severe
☐	☐	☐	☐	☐	☐

MUSCLES AND JOINTS

3. Have you during the last <u>year</u> been troubled with pain and/or stiffness in muscles or joints, that has lasted for <u>minimum 3 months continuously?</u> (If no, proceed to HEADACHE)

4. <u>If yes</u>: Where have you had these symptoms? (Mark one or more boxes)

Yes	No
☐	☐

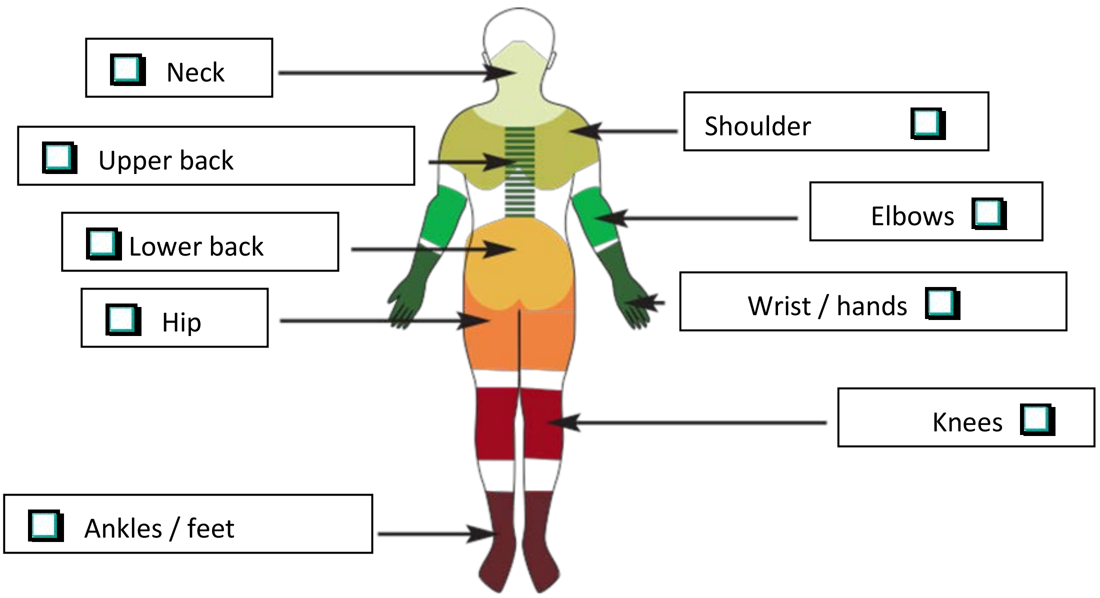

5. Have you been troubled in both right and left half of the body?

Yes	No
☐	☐

6. Has the pain hindered you in performing daily activities?

	Yes	No
During work……………………………………..	☐	☐
In leisure time ……………………………….	☐	☐

HEADACHE

7. Have you been troubled with headache the last year? Yes ☐ No ☐
   (If no, proceed to SLEEP)
   If yes: What type of headache: Migraine ☐ Other headache ☐

8. Ca. number of days per month with headache:
   Less than 1 day ☐ 1-6 days ☐ 7-14 days ☐ More than 14 days ☐

9. How strong is the headache usually?
   - Mild (does not hamper activity) ☐
   - Moderate (hampers activity) ☐
   - Strong (hinders activity) ☐

10. Is the headache usually characterised or accompanied by:
(Put one mark per row)

	Yes	No
Throbbing/pulsatory pain?	☐	☐
Pressing pain?..........	☐	☐
One-sided pain (right or left)?..........	☐	☐
Worsens with moderate physical activity?	☐	☐
Nausea and/or vomiting?..........	☐	☐
Photophobia or phono phobia?	☐	☐

11. Before or during the headache, do you sometimes have:
(Put one mark per row)

	Yes	No
Distorted vision? (jagged lines, flickering, blurred vision, flash of light)	☐	☐
Numbness in one side of the face or in the hand?	☐	☐

12. Indicate how many days you have been absent from work or school the last month due to headache: ☐ days

SLEEP

13. How often has it happened in the course of the last 3 months that you:	Never	Seldom	Occationally (some times per month)	Mostly (several times per week)	Always (daily)
Snore loudly and bothersome?	☐	☐	☐	☐	☐
Get breathing stops while sleeping?..........	☐	☐	☐	☐	☐
Find it difficult to fall asleep at night?	☐	☐	☐	☐	☐
Wake up repeatedly at night?	☐	☐	☐	☐	☐

Wake up too early and can't get back to sleep?	☐	☐	☐	☐	☐
Feel sleepy during the day?	☐	☐	☐	☐	☐
Wake up with a headache?	☐	☐	☐	☐	☐
Get discomfort or tingling in legs?	☐	☐	☐	☐	☐
Have unintended sleep episodes ("head bobbing") at work or school?…………..	☐	☐	☐	☐	☐
Have unintended sleep episodes ("head bobbing") in your free time?………………………………..	☐	☐	☐	☐	☐

14. How often are you bothered by insomnia?

Never, or a few times a year ………….. ☐ About once a week ………………… ☐

1-2 times a month …………………… ☐ More than once a week.…………… ☐

15. Have you during the last year been bothered by insomnia affecting your ability to work? Yes ☐ No ☐

16. Have you during the last month had problems falling asleep? *Mark only one alternative*

Almost every night ……………….................... ☐ Occationally ……………………..... ☐

Often ………………….. ….………………….. ☐ Never ………………………………. ☐

17. Have you during the last month woken up too early without getting back to sleep? *Mark only one alternative*

Almost every night ……………….................... ☐ Occationally ……………………..... ☐

Often ………………….. ….………………….. ☐ Never ………………………………. ☐

18. When do you normally go to bed to sleep?

During working week at ________ o'clock

In your free time at ________ o'clock

19. When do you normally wake up? (final awakening)

During working week at ________ o'clock

In your free time at ________ o'clock

20. For how long do you stay awake (in bed) before you fall asleep? (Number of minutes)

During working week ________ minutes

In your free time ________ minutes

21. How much sleep do you need? (Number of hours) ____ timer

22. How many hours do you sleep on average per 24? (night + daytime sleep) ____ timer

23. Do you think you get sufficient/enough sleep?

Yes, nearly always ☐ Yes, often ☐

Seldom or nearly never ☐ Don't know ☐
