# Supplementary material for: Maternal serum retinol, 25(OH)D and 1,25(OH)2D concentrations during pregnancy and peak bone mass and trabecular bone score in adult offspring at 26-year follow-up
Source: PLoS One. 2019 Sep 26;14(9):e0222712. doi: 10.1371/journal.pone.0222712 (PMC6762137; doi:10.1371/journal.pone.0222712)
Supplement: S4 File — (PDF) [file pone.0222712.s007.pdf]

DIET AN EATING HABITS

1. How often do you typically eat these foods? *(select one alternative per line)*

	0-3 times per month	1-3 times per month	4-6 times per week	1 time per day	2 times or more per day
Fruit/berries	☐	☐	☐	☐	☐
Vegetables	☐	☐	☐	☐	☐
Chocolate/sweets	☐	☐	☐	☐	☐
Boiled potatoes	☐	☐	☐	☐	☐
Pasta/rice	☐	☐	☐	☐	☐
Sausages/burgers	☐	☐	☐	☐	☐
Fatty fish (salmon, trout, herring, mackerel, redfish as spread/dinner)	☐	☐	☐	☐	☐

2. Do you use the following food supplements? *(select one alternative for each supplement)*

	Yes, daily	Occasionally	No
Cod-liver oil	☐	☐	☐
Omega-3 capsules	☐	☐	☐
Vitamin-and/or mineral supplements	☐	☐	☐

3. How <u>many glasses</u> do you typically drink of the following? *(1/2 liter = 3 glasses, select one alternative per line)*

	Seldom or never	1-6 gl. per week	1 gl. per day	2-3 gl. per day	4 gl. or more per day
Water, mineral water and the like	☐	☐	☐	☐	☐
Whole milk (sweet/sour)	☐	☐	☐	☐	☐
Other milk (sweet/sour)	☐	☐	☐	☐	☐
Soft drink/soda with sugar	☐	☐	☐	☐	☐
Soft drink/soda without sugar	☐	☐	☐	☐	☐
Juice or nectar	☐	☐	☐	☐	☐

4. How many cups of coffee/tea do you drink <u>per 24 hours</u>?
(Insert 0 for the type of coffee/tea that you do not drink daily) Number of cups:

	Number of cups
Tea	__ __
Bioled coffee/cafetiére/coffee capsules / coffee machine at Café	__ __
Drip coffee/filter coffee	__ __
Coffee powder (instant coffee)	__ __

5. How many cups of coffee do you have in the evening *(after 6 pm)*?

Number of cups: __ __

6. Below is a list of items that apply to eating habits *(select one alternative per line)*

	Never	Seldom	Often	Always
When I first have started eating, it can be hard to stop	☐	☐	☐	☐
I throw up after I have eaten	☐	☐	☐	☐
I spend too much time to think about food	☐	☐	☐	☐
I feel that food controls my life	☐	☐	☐	☐
When I eat, I cut the food into small pieces	☐	☐	☐	☐
I use longer than others on a meal	☐	☐	☐	☐
Other people think that I am too thin	☐	☐	☐	☐
I feel that others are pushing me to eat	☐	☐	☐	☐

7. Would you say about yourself that you are: *(check one box)*

Very thick	A bit thick	Much like others	Rather thin	Very thin
☐	☐	☐	☐	☐

8. Are you trying to lose weight? (check one box*)*

☐ No, my weight is appropriate ☐ No, but I need to lose weight ☐ Yes
