# Supplementary material for: Maternal serum retinol, 25(OH)D and 1,25(OH)2D concentrations during pregnancy and peak bone mass and trabecular bone score in adult offspring at 26-year follow-up
Source: PLoS One. 2019 Sep 26;14(9):e0222712. doi: 10.1371/journal.pone.0222712 (PMC6762137; doi:10.1371/journal.pone.0222712)
Supplement: S5 File — (PDF) [file pone.0222712.s008.pdf]

ABOUT MOTOR SKILLS AND PHYSICAL ACTIVITY

1. To what extent do you experience that you have motor problems?

☐ Not at all ☐ To a small extent ☐ To some extent ☐ To a large extent ☐ To a very large extent

2. To what extent do the motor problems affect activities in everyday life?

☐ Not applicable (do not have motor difficulties)

At work:
☐ Not at all ☐ To a small extent ☐ To some extent ☐ To a large extent ☐ To a very large extent

In leisure time:
☐ Not at all ☐ To a small extent ☐ To some extent ☐ To a large extent ☐ To a very large extent

3. Would you say that these problems are mainly:

☐ Fine motor problems ☐ Gross motor problems ☐ Both

4. Have you been receiving help for your motor problems? ☐ No ☐ Yes

5. If yes, of whom?

☐ Physiotherapist ☐ Occupational therapist ☐ General practitioner ☐ Health nurse ☐ Nurse

☐ Educational Psychological Service

What kind of help? ____

Was your experience that this helped? ☐ No ☐ Yes

6. How physically demanding is your work/education?

☐ I am not working/studying

☐ My work/education is mainly sitting work and I do not move around much during working hours (e.g. a watchmaker's, radio mechanic's, industry dressmaker's work, or office work at your desk)

☐ I move pretty much in my work/education, but I need not lift or carry heavy objects (e.g. a foreman or a store salesperson's work, light industry work, office work that requires moving around)

☐ In my work/education I have to walk and lift a lot or often walk up stairs or hills (e.g. a carpenter's or caretaker's work, machine repair shop work – or similar heavier industry work)

☐ My work/education is heavy bodywork, where I have to lift and carry heavy objects, dig, shovel or chop etc. (e.g. forestry, heavy agricultural work, heavy construction or industrial work)

7. How many hours a week do you work/study? __ __ hours __ __ minutes

8. How many minutes do you spend walking, biking or move in other physical strenuous ways under travel to and from work/study place? *Include the overall time for journey to and from work/study place.*

- ☐ I do not work/study, or I travel all the way to work/study place with motor vehicle
- ☐ Less than 15 minutes per day
- ☐ 15-29 minutes per day
- ☐ 30-44 minutes per day
- ☐ 45-59 minutes per day
- ☐ 1 hour or more per day

9. How many hours are you sitting on average on a weekday?

During work/study in office or the like	__ __ t __ __ min
At home in front of the tv, video, or data	__ __ t __ __ min
In a vehicle	__ __ t __ __ min
Other places	__ __ t __ __ min

10. Outside of school/work: How often do you engage yourself in sports or other forms of physical exercise so much that you are out of breath and/or sweaty? *Do not include exercise during travel to/from work.*

- ☐ Not at all
- ☐ Less often than 1 time a month
- ☐ 1-2 times a month
- ☐ Ca. 1 time a week
- ☐ 2-3 times a week
- ☐ 4-5 times a week
- ☐ About daily

11. For how long do you engage in sports or physical activity each time in your leisure time?

- ☐ I do not exercise in my leisure time
- ☐ Less than 30 minutes
- ☐ 30-59 minutes
- ☐ 1- <2 hours

☐ 2 hours or more

12. How intense would you rate your leisure-time exercise?
Check the alternative that best describes the intensity..

☐ Walking

☐ Alternate between walking and easy running

☐ Easy running (jogging)

☐ Energetic running (interval training)

13. On a scale from 6 to 20, where 6 is not strenuous and 20 is maximum strenuous: How heavy is the burden usually when you do sports or exercise?
The following provides an explanation and description of the load by some of the numbers.

☐ 6 No effort. You notice no effort at all, for example no muscle fatigue, breathlessness or dyspnea

☐ 7 Very, very easy

☐ 8

☐ 9 Very easy. Like a short hike in your own pace

☐ 10

☐ 11 Quite easy

☐ 12

☐ 13 A little tiring. You can continue without major problems

☐ 14

☐ 15 Tiring. You're tired, but can still continue

☐ 16

☐ 17 Very tiring. A very large strain. You can continue, but need to exert yourself hard, and feel very tired

☐ 18

☐ 19 Extremely tiring. For most people this is equivalent to the largest effort they have ever experienced

☐ 20 A maximum of strenuous

14. What kind of exercise/sports are you doing? Mention all the activities:

____

____

15. How many minutes per day do you spend walking, biking or otherwise exercising in your leisure time? ***(in household work and gardening, repair work, tiding/cleaning)?***
(Do not count the time that you are active at work, or in travel to and from work, or the time off-hours that you do sports/exercise)

☐ Less than 15 minutes per day

☐ 15-29 minutes per day

☐ 30-44 minutes per day

☐ 45-59 minutes per day

☐ 1 hour or more per day

16. Do you have <u>at least 30 minutes</u> of daily physical activity at work and/or in your leisure time?

☐ No ☐ Yes

17. How do your rate your physical shape nowadays?

☐ Very good ☐ Fairly good ☐ Appropriate ☐ Fairly bad ☐ Very bad

18. Have you ever tried anabolic steroids or other doping agents? *(Check one box)*

☐ No ☐ Yes

If yes, how old were you the first time? _____ Years

19. Have you used anabolic steroids or other doping agents last 3 months?

☐ No ☐ Yes
