# Supplementary material for: Maternal serum retinol, 25(OH)D and 1,25(OH)2D concentrations during pregnancy and peak bone mass and trabecular bone score in adult offspring at 26-year follow-up
Source: PLoS One. 2019 Sep 26;14(9):e0222712. doi: 10.1371/journal.pone.0222712 (PMC6762137; doi:10.1371/journal.pone.0222712)
Supplement: S6 File — (PDF) [file pone.0222712.s009.pdf]

QUESTIONS FOR WOMEN

1. **Do you have a steady boyfriend?** ☐ No ☐ No, not now, but previously ☐ Yes

   Are you satisfied with your love life? ☐ No ☐ No, not now, but previously ☐ Yes

2. **How old were you when you had your first menstrual period?**
   I was ____ years and _____ months

3. **Do you have regular menstruation?**
   ☐ No ☐ Yes ___ weeks between each menstruation

4. **How many menstrual periods have you had <u>the last 12 months</u>?** _____ number

5. **How many days since the first day of the last mentruation?** _____ days

6. **Have you ever used birth control pills?**

   ☐ No ☐ Yes, I used previously ☐ Yes, I am presently using

   If yes:
   How old were you the first time you used birth control pills? _______ years

   For how long have you used birth control pills in all? _______ years in all

7. **Have you ever used any of the following contraceptives?**

	No	Yes, I used previously	Yes, I am presently using
P-syringe/P-Rod/Mini-pill	☐	☐	☐
P-ring/P-patches	☐	☐	☐
Spiral without hormones	☐	☐	☐ Spiral with
hormones	☐	☐	☐
Diaphragm	☐	☐	☐
Condom	☐	☐	☐
Interrupted intercourse	☐	☐	☐
Sterilization	☐	☐ Yes, myself	☐ Yes, partner

8. **Have you ever had sexual intercourse?** ☐ No ☐ Yes

   If yes, how old were you the first time ______ years

9. **Have you ever used morning-after pill?** ☐ No ☐ Yes ☐ Several times

   If yes, how old were you the first time ______ years

10. Have you ever been pregnant? ☐ No ☐ Yes

If yes, how old were you when this happened?

1. time	2. time	3. time
______ years	______ years	______ years

Did you want this pregnancy? *(Check for each pregnancy)*

	1. time	2. time	3. Time
	☐ No<br>☐ Yes<br>☐ Don't know	☐ No<br>☐ Yes<br>☐ Don't know	☐ No<br>☐ Yes<br>☐ Don't know
Did you have an induced abortion? Check only if YES	☐	☐	☐

11. Are you pregnant now? ☐ No ☐ Yes If yes, number of weeks: ______

12. Do you have children? ☐ No ☐ Yes If yes, how many: ______

If yes:

How old were you when you became a mom for the first time? I was ___ years + ___ months

Child 1: Birth weight: ______ g Length: _____ cm Gestational age: ____ weeks ____days

☐ Born preterm (>3 weeks before term) ☐ Born at term (week 37 - 42) ☐ Born after week 42

Child 2: Birth weight: ______ g Length: _____ cm Gestational age: ____ weeks ____days

☐ Born preterm (>3 weeks before term) ☐ Born at term (week 37 - 42) ☐ Born after week 42

Child 3: Birth weight: ______ g Length: _____ cm Gestational age: ____ weeks ____days

☐ Born preterm (>3 weeks before term) ☐ Born at term (week 37 - 42) ☐ Born after week 42

13. How long is it since the last birth? ____ years and _____ months

14. Have you menstruated after the last birth? ☐ No ☐ Yes

15. Are you breastfeeding now? ☐ No ☐ Yes
