# Supplementary material for: Maternal serum retinol, 25(OH)D and 1,25(OH)2D concentrations during pregnancy and peak bone mass and trabecular bone score in adult offspring at 26-year follow-up
Source: PLoS One. 2019 Sep 26;14(9):e0222712. doi: 10.1371/journal.pone.0222712 (PMC6762137; doi:10.1371/journal.pone.0222712)
Supplement: S7 File — (PDF) [file pone.0222712.s010.pdf]

QUESTIONS FOR MEN

1. Do you have a steady girlfriend? ☐ No ☐ No, not now, but previously ☐ Yes

Are you satisfied with your love life? ☐ No ☐ No, not now, but previously ☐ Yes

2. Have you ever had sexual intercourse? ☐ No ☐ Yes

If yes, how old were you the first time ______ years

3. Has a woman ever been pregnant together with you? ☐ No ☐ Yes

If yes, how old were you when this happened? 1. time ______ years 2. time ______ years 3. time ______ years

Did you want this pregnancy? *(Check for each pregnancy)*

	1. time	2. time	3. Time
	☐ No<br>☐ Yes<br>☐ Don't know	☐ No<br>☐ Yes<br>☐ Don't know	☐ No<br>☐ Yes<br>☐ Don't know
Did she have an induced abortion? Check only if YES	☐	☐	☐

4. Do you have children? ☐ No ☐ Yes If yes, how many: ______

If yes:

How old were you when you became a mom for the first time? I was ___ years + ___ months

Child 1: Birth weight: ______ g Length: _____ cm Gestational age: ____ weeks ____days

☐ Born preterm (>3 weeks before term) ☐ Born at term (week 37 - 42) ☐ Born after week 42

Child 2: Birth weight: ______ g Length: _____ cm Gestational age: ____ weeks ____days

☐ Born preterm (>3 weeks before term) ☐ Born at term (week 37 - 42) ☐ Born after week 42

Child 3: Birth weight: ______ g Length: _____ cm Gestational age: ____ weeks ____days

☐ Born preterm (>3 weeks before term) ☐ Born at term (week 37 - 42) ☐ Born after week 42
