# Supplementary material for: Maternal serum retinol, 25(OH)D and 1,25(OH)2D concentrations during pregnancy and peak bone mass and trabecular bone score in adult offspring at 26-year follow-up
Source: PLoS One. 2019 Sep 26;14(9):e0222712. doi: 10.1371/journal.pone.0222712 (PMC6762137; doi:10.1371/journal.pone.0222712)
Supplement: S8 File — (PDF) [file pone.0222712.s011.pdf]

BAKGRUNNSINFORMASJON

Kjønn: 0 ☐ **Kvinne** 1 ☐ **Mann** **Etnisk bakgrunn** ____

Hvem bor du sammen med nå? (velg kun ett alternativ)

1 ☐ Foreldre/foresatte	2 ☐ Søsken	3 ☐ Øvrige familie
4 ☐ Alene på hybel	5 ☐ Alene i leilighet	6 ☐ Bofellesskap/kollektiv
7 ☐ Partner/ektefelle	8 ☐ Bolig med tilsyn	9 ☐ Annet – beskriv: ______________

Ev. tilleggsopplysninger: ____

Sivil status:

1 ☐ Gift 2 ☐ Samboer 3 ☐ Skilt 4 ☐ Enslig

SØSKEN: Kjønn og alder

0 ☐ Kvinne	1 ☐ Mann	________ år	0 ☐ Kvinne	1 ☐ Mann	________ år
0 ☐ Kvinne	1 ☐ Mann	________ år	0 ☐ Mann	1 ☐ Mann	________ år

Hvis flere: Noter ____

UTDANNING, ARBEID OG ØKONOMI

1 ☐ Under utdanning 2 ☐ I arbeid 3 ☐ Trygdet 4 ☐ Arbeidsledig

Ev. beskriv: ____

<u>FULLFØRT</u> utdanning:

1 ☐ Barneskole (1.-7. klasse)	9 ☐ Fagskoleutdanning 1-2 år: ____________________
2 ☐ Ungdomsskole (8.-10. klasse)	10 ☐ Bachelor: ________________________________
3 ☐ Videregående trinn 1 (Vg/VK1)	11 ☐ Master:________________________________
4 ☐ Videregående trinn 2 (Vg/VK2)	12 ☐ Embetsstudie: ____________________________
5 ☐ Videregående Vg3 m/generell studiekompetanse	13 ☐ Doktorgrad (PhD): _________________________
6 ☐ Videregående påbygging m/generell studiekomp.	14 ☐ Ukjent/annet
7 ☐ Opplæring i bedrift, 2-årig (lærling) m/fagbrev	Ev. beskriv: __________________________________
8 ☐ Folkehøgskole	**Ble utdanningen fullført på normert tid?**
	0 ☐ Nei 1 ☐ Ja

Nåværende yrke: ____

<u>Din</u> samlede inntekt siste år:

1 ☐ Under 100.000	2 ☐ 100-199.000	3 ☐ 200-349.000
4 ☐ 350-549.000	5 ☐ 550-749.000	6 ☐ ≥ 750.000

Hvordan vil du beskrive din økonomi:

1 ☐ Svært dårlig 2 ☐ Dårlig 3 ☐ Middels 4 ☐ God 5 ☐ Svært god

Er du helt avhengig av økonomisk bidrag fra din partner:	0 ☐ Nei	1 ☐ Ja
Er du helt avhengig av økonomisk bidrag fra dine foreldre/øvrige familie:	0 ☐ Nei	1 ☐ Ja

SOMATISK OG PSYKIATRISK SYKEHISTORIE

Hvem er din fastlege? ____

Har du noen sykdommer/plager som trenger regelmessig oppfølging eller kontroll? (somatiske sykdommer, f.eks. astma, sukkersyke, epilepsi etc.)

0 ☐ Nei 1 ☐ Ja Evt. hvilke(n) ____________________

Får du slik oppfølging nå? 0 ☐ Nei 1 ☐ Ja Presiser for hva: ____________________

Hos hvem (navn på lege/andre, evt. avd./sykehus): ______________ ____________________

Tar du noen medisiner (inkludert astmainhalasjon) *nå?* 0 ☐ Nei 1 ☐ Ja

Medikamentnavn og dose: ____________________ Dose: ______ Daglig: ____ (kryss) Etter behov: _____

Medikamentnavn og dose: ____________________ Dose: ______ Daglig: ____ (kryss) Etter behov: _____

Ev. tidligere: 0 ☐ Nei 1 ☐ Ja

Medikamentnavn og dose: ____________________ Dose: _______ Fra ___.___.___ Til ___.___.___

Medikamentnavn og dose: ____________________ Dose: _______ Fra ___.___.___ Til ___.___.___

Noen gang innlagt på sykehus? 0 ☐ Nei 1 ☐ Ja For hva: ____________________

Alvorlige fysiske skader? 0 ☐ Nei 1 ☐ Ja Hva slags: ____________________

Hodeskader? (Angi om ble bevisstløs): 0 ☐ Nei 1 ☐ Ja Hva slags: ____________________

Andre somatiske problemer nå/før: 0 ☐ Nei 1 ☐ Ja Hvilke: ____________________

Hørsel

Redusert hørsel? 0 ☐ Nei 1 ☐ Ja Hvis **ja**, hvilket avvik? ____________________

Syn

Redusert syn? 0 ☐ Nei 1 ☐ Ja

Bruker briller? 0 ☐ Nei 1 ☐ Ja – til lesing eller pc 2 ☐ Fast

Hvis **ja**, hvorfor? (årsak til synsreduksjon) ____

Lærevansker

Har du lærevansker? 0 ☐ Nei 1 ☐ Ja

Hvis **ja**, hvilke?

1 ☐ Lese/skrivevansker

2 ☐ Regnevansker

3 ☐ Andre, beskriv ____________________

Hvilken utredning har blitt utført? ____

Hvilken hjelp har du fått? ____

Motoriske vansker

Har du motoriske vansker (føler deg klosset, koordineringsvansker etc.)? 0 ☐ Nei 1 ☐ Ja

Hvis **ja**, beskriv: ____

____

PSYKISKE PLAGER

Nå: 0 ☐ Nei 1 ☐ Ja

____

____

____

Tidligere: 0 ☐ Nei 1 ☐ Ja

____

____

____

Får du hjelp for psykiske plager nå? 0 ☐ Nei 1 ☐ Ja

Har du fått hjelp for psykiske plager tidligere? 0 ☐ Nei 1 ☐ Ja

Hvis **ja**, av hvem? 1 ☐ psykolog 2 ☐ psykiater 3 ☐ fastlege 4 ☐ helsesøster 5 ☐ sykepleier 6 ☐ PPT

Poliklinisk behandling 0 ☐ Nei 1 ☐ Ja Alder: ___ år

Dagpasient/ambulant team 0 ☐ Nei 1 ☐ Ja Alder: ___ år

Innleggelse 0 ☐ Nei 1 ☐ Ja Alder: ___ år

Antall innleggelser på psykiatrisk sykehus ____ Ev. navn på sykehus: ______________________

Medisinliste (Psykofarmaka) NÅ:

1 Antipsykotika ____________________ 0 ☐ Nei 1 ☐ Ja
____

4 Stimulantia ____________________ 0 ☐ Nei 1 ☐ Ja
____

2 Antidepressiva ____________________ 0 ☐ Nei 1 ☐ Ja
____

5 Stemn.stabil.____________________ 0 ☐ Nei 1 ☐ Ja
____

3 Sedativa ____________________ 0 ☐ Nei 1 ☐ Ja
____

6 Annet ____________________ 0 ☐ Nei 1 ☐ Ja
____

Kommentar etter innledning: ____
