# Supplementary material for: Maternal serum retinol, 25(OH)D and 1,25(OH)2D concentrations during pregnancy and peak bone mass and trabecular bone score in adult offspring at 26-year follow-up
Source: PLoS One. 2019 Sep 26;14(9):e0222712. doi: 10.1371/journal.pone.0222712 (PMC6762137; doi:10.1371/journal.pone.0222712)
Supplement: S9 File — (PDF) [file pone.0222712.s012.pdf]

SOMATISK UNDERSØKELSE

1. Har du (hatt) høyt blodtrykk? Tidligere: ☐ Nei ☐ Ja Nå: ☐ Nei ☐ Ja

2. Går du til spesialist for høyt blodtrykk NÅ? ☐ Nei ☐ Ja

3. Tar du blodtrykksmedisiner NÅ? ☐ Nei ☐ Ja

---

4. Har du (hatt) hjerteproblemer? Tidligere: ☐ Nei ☐ Ja Nå: ☐ Nei ☐ Ja

5. Går du til spesialist for hjerteproblemer NÅ? ☐ Nei ☐ Ja

6. Tar du medisiner for hjerteproblemer NÅ? ☐ Nei ☐ Ja

---

7. Har du (hatt) lungeproblemer?
a) Astma: Tidligere: ☐ Nei ☐ Ja Nå: ☐ Nei ☐ Ja
b) Bronkitt: Tidligere: ☐ Nei ☐ Ja Nå: ☐ Nei ☐ Ja
c) Lungebetennelse: Tidligere: ☐ Nei ☐ Ja Nå: ☐ Nei ☐ Ja

8. Har du vært innlagt på sykehuset for lungeproblemer? ☐ Nei ☐ Ja

9. Går du til spesialist for lungeproblemer NÅ? ☐ Nei ☐ Ja

10. Tar du medisiner for lungesykdom NÅ? ☐ Nei ☐ Ja

11. Røykt/røyker? Tidligere: ☐ Nei ☐ Ja Ant. sigaretter dgl: ____ Hvor lenge (år): ____
Nå: ☐ Nei ☐ Ja Ant. sigaretter dgl: ____ Hvor lenge (år): ____

12. Snus? Tidligere: ☐ Nei ☐ Ja Ant. priser dgl: ____ Hvor lenge (år): ____
Nå: ☐ Nei ☐ Ja Ant. priser dgl: ____ Hvor lenge (år): ____

---

13. Har du diabetes? ☐ Nei ☐ Ja ☐ Diabetes 1 ☐ Diabetes 2

14. Hvis ja, når fikk du diabetes? _____ år gammel

15. Tar du medisiner for diabetes? ☐ Nei ☐ Ja, tabletter ☐ Ja, insulin (sprøyter)

---

16. Har du hatt beinbrudd? ☐ Nei ☐ Ja Hvor gammel var du? ____ år (hvis flere, noter alle)

17. Hvis ja, lokalisasjon (hvis flere, noter alle): ___________________________________

18. Hvis ja, hvordan skjedde det (beskriv): _______________________________________
Lavenergibrudd ☐ Traumatisk brudd ☐

---

19. Har du (hatt) epilepsi? Tidligere: ☐ Nei ☐ Ja Nå: ☐ Nei ☐ Ja

20. Går du til spesialist for epilepsi NÅ? ☐ Nei ☐ Ja

21. Tar du medisiner for epilepsi NÅ? ☐ Nei ☐ Ja

---

22. Har du cerebral parese? ☐ Nei ☐ Ja

23. Andre sykdommer (f.eks leddplager/reumatiske plager, annet): _________________

UNDERSØKELSE: Undersøker: _____________ Dato: __________

Blodtrykk i sittende på høyre arm etter 5 min hvile 3 ganger med 2 min mellomrom:

	Systolisk BT	Diastolisk BT	MAP	Puls
1. måling				
2. måling				
3. måling				

Hodeomkrets: _____ cm (en desimal)

Overarmslengde: Høyre _____ cm (en desimal) Venstre _____ cm (en desimal)

Overarmsomkrets: Høyre _____ cm (en desimal) Venstre _____ cm (en desimal)

Midjeomkrets over navle: _____ cm (en desimal)

Midjeomkrets mellom hoftekam og nederste ribbe: _____ cm (en desimal)

Hofteomkrets: _____ cm (en desimal)

Hudfolder: 1. Venstre triceps hudfold 1. måling: _____ mm (en desimal)
2. måling: _____ mm (en desimal)

2. Venstre subscapulære hudfold 1. måling: _____ mm (en desimal)
2. måling: _____ mm (en desimal)

INTERVJU: Om motorikk og fysisk aktivitet

SPØRRESKJEMA: Sjekk at spørreskjema er fullstendig utfylt:

1. Om kosthold og spisevaner
2. Spørsmål for kvinner og menn – NB! Fødselsvekt og lengde på deres barn
3. Smerter – hodepine – søvn
4. Fatigue Severity Scale
