# Supplementary material for: Maternal serum retinol, 25(OH)D and 1,25(OH)2D concentrations during pregnancy and peak bone mass and trabecular bone score in adult offspring at 26-year follow-up
Source: PLoS One. 2019 Sep 26;14(9):e0222712. doi: 10.1371/journal.pone.0222712 (PMC6762137; doi:10.1371/journal.pone.0222712)
Supplement: S10 File — (PDF) [file pone.0222712.s013.pdf]

SMERTER

1. Har du kroppslige smerter nå som har vart i <u>mer enn 6 måneder</u>?

Ja	Nei
☐	☐

2. Hvor sterke kroppslige smerter har du hatt i løpet av <u>de siste 4 uker</u>?

Ingen	Meget svake	Svake	Moderate	Sterke	Meget sterke
☐	☐	☐	☐	☐	☐

MUSKLER OG LEDD

3. Har du i løpet av det <u>siste året</u> vært plaget med smerter og/eller stivhet i muskler og ledd, som har vart i <u>minst 3</u> <u>måneder sammenhengende</u>? (Hvis nei, gå til HODEPINE)

4. <u>Hvis ja</u>: Hvor har du hatt disse plagene? (Sett ett eller flere kryss)

Ja	Nei
☐	☐

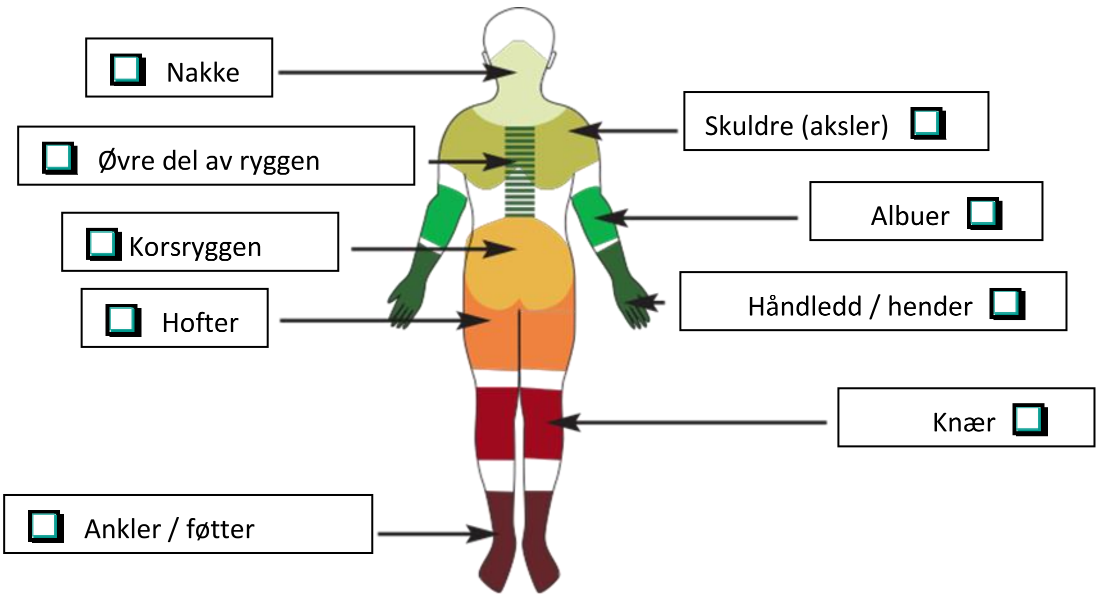

5. Har du vært plaget både i høyre og venstre kroppshalvdel?

Ja	Nei
☐	☐

6. Har plagene hindret deg i å utføre daglige aktiviteter?

	Ja	Nei
I arbeid	☐	☐
I fritid	☐	☐

HODEPINE

7. Har du vært plaget av hodepine det siste året? Ja ☐ Nei ☐
(Hvis nei, gå til SØVN)
Hvis ja: Hva slags hodepine?: Migrene ☐ Annen hodepine ☐

8. Omtrent antall dager pr. måned med hodepine:
Mindre enn 1 dag ☐ 1-6 dager ☐ 7-14 dager ☐ Mer enn 14 dager ☐

9. Hvor sterk er hodepina vanligvis?
Mild (hemmer ikke aktivitet) ☐
Moderat (hemmer aktivitet) ☐
Sterk (forhindrer aktivitet) ☐

10. Er hodepina vanligvis preget av eller ledsaget av:
(Sett ett kryss pr. linje)

	Ja	Nei
Bankende/dunkende smerte?	☐	☐
Pressende smerte?...	☐	☐
Ensidig smerte (høyre eller venstre)?..	☐	☐
Forverring ved moderat fysisk aktivitet?	☐	☐
Kvalme og/eller oppkast?...	☐	☐
Lys- og lydskyhet?	☐	☐

11. Før eller under hodepina; kan du ha forbigående:
(Sett ett kryss pr. linje)

	Ja	Nei
Synsforstyrrelse? (takkede linjer, flimring, tåkesyn, lysglimt) …………………	☐	☐
Nummenhet i halve ansiktet eller i handa?	☐	☐

12. Angi hvor mange dager du har vært borte fra arbeid eller skole siste måned på grunn av hodepine: ☐☐ dager

SØVN

13. Hvor ofte har det hendt i løpet av de siste 3 måneder at du:

	Aldri	Sjelden	Iblant (noen ganger per måned)	For det meste (flere ganger per uke)	Alltid (hver dag)
Snorker høyt og sjenerende?	☐	☐	☐	☐	☐
Får pustestopp når du sover?	☐	☐	☐	☐	☐
Har vanskelig for å sovne om kvelden?	☐	☐	☐	☐	☐

Våkner gjentatte ganger om natta?	☐	☐	☐	☐	☐
Våkner for tidlig og får ikke sove igjen? …….	☐	☐	☐	☐	☐
Kjenner deg søvnig om dagen?	☐	☐	☐	☐	☐
Våkner med hodepine?	☐	☐	☐	☐	☐
Får ubehag, kribling eller mauring i bein? ….	☐	☐	☐	☐	☐
Har utilsiktede søvnepisoder ("hodet dupper") på arbeid eller skole? ………………………...	☐	☐	☐	☐	☐
Har utilsiktede søvnepisoder ("hodet dupper") i fritiden? ………………………………………..	☐	☐	☐	☐	☐

14. Hvor ofte er du plaget av søvnløshet?

Aldri eller noen få ganger i året …………..	☐	Omtrent 1 gang i uka ……………..	☐
1-2 ganger i måneden ……………………	☐	Mer enn en gang i uka ..…………..	☐

15. Har du siste året vært plaget av søvnløshet slik at det har gått ut over arbeidsevnen din?

Ja ☐ Nei ☐

16. Har du i løpet av den siste måned hatt innsovningsproblemer? *Bare ett kryss*

Nesten hver natt ………………....................	☐	Av og til ………………………….....	☐
Ofte ………………….. ……………………..	☐	Aldri ………………………………..	☐

17. Har du i løpet av den siste måned våknet for tidlig og ikke fått sove igjen? Bare ett kryss

Nesten hver natt ………………....................	☐	Av og til ………………………….....	☐
Ofte ………………….. ……………………..	☐	Aldri ………………………………..	☐

18. Når går du normalt til sengs for å sove?

I arbeidsuken klokken ________

I fritiden klokken ________

19. Når våkner du normalt opp? (endelig oppvåkning)

I arbeidsuken klokken ________

I fritiden klokken ________

20. Hvor lenge ligger du våken før du sovner? (Antall minutter)

I arbeidsuken ________ minutter

I fritiden ________ minutter

21. Hvor mye søvn trenger du? (Antall timer) _________ timer

22. Hvor mange timer sover du i gjennomsnitt pr døgn? (natt + dag-søvn) ____ timer

23. Synes du at du sover tilstrekkelig/nok?

Ja, nesten alltid	☐	Ja, ofte	☐
Sjelden eller nesten aldri	☐	Vet ikke	☐
