# Supplementary material for: Maternal serum retinol, 25(OH)D and 1,25(OH)2D concentrations during pregnancy and peak bone mass and trabecular bone score in adult offspring at 26-year follow-up
Source: PLoS One. 2019 Sep 26;14(9):e0222712. doi: 10.1371/journal.pone.0222712 (PMC6762137; doi:10.1371/journal.pone.0222712)
Supplement: S11 File — (PDF) [file pone.0222712.s014.pdf]

OM KOSTHOLD OG SPISEVANER

1. Hvor ofte spiser du vanligvis disse matvarene? *(Sett ett kryss pr. linje)*

	0-3 ganger pr. mnd.	1-3 ganger pr. uke	4-6 ganger pr. uke	1 gang pr. dag	2 ggr el mer pr. dag
Frukt/bær	☐	☐	☐	☐	☐
Grønnsaker	☐	☐	☐	☐	☐
Sjokolade/smågodt	☐	☐	☐	☐	☐
Kokte poteter	☐	☐	☐	☐	☐
Pasta/ris	☐	☐	☐	☐	☐
Pølser/hamburgere	☐	☐	☐	☐	☐
Fet fisk (laks, ørret, sild, makrell, uer som pålegg/middag)	☐	☐	☐	☐	☐

2. Bruker du følgende kosttilskudd? *(sett ett kryss for hvert kosttilskudd)*

	Ja, daglig	Av og til	Nei
Tran	☐	☐	☐
Omega-3-kapsler	☐	☐	☐
Vitamin- og/eller mineraltilskudd	☐	☐	☐

3. Hvor <u>mange glass</u> drikker du vanligvis av følgende? *(1/2 liter = 3 glass, sett ett kryss pr. linje)*

	Sjelden el. aldri	1-6 gl. pr. uke	1 gl. pr. dag	2-3 gl. pr. dag	4 gl. el. mer pr. dag
Vann, farris og lignende	☐	☐	☐	☐	☐
Helmelk (søt/sur)	☐	☐	☐	☐	☐
Annen melk (søt/sur)	☐	☐	☐	☐	☐
Brus/saft med sukker	☐	☐	☐	☐	☐
Brus/saft uten sukker	☐	☐	☐	☐	☐
Juice eller nektar	☐	☐	☐	☐	☐

4. Hvor mange kopper kaffe/te drikker du pr. døgn?
(Sett 0 på type kaffe/te som du ikke drikker daglig) Antall kopper:

Te __ __

Kokekaffe/presskanne/kaffekapsler/kaffemaskin på kafé __ __

Traktekaffe/filterkaffe __ __

Kaffepulver (instant kaffe) __ __

5. Hvor mange kopper kaffe drikker du om kvelden *(etter kl. 18)* Antall kopper: __ __

6. Nedenfor er en liste over ting som gjelder spisevaner (*Sett ett kryss for hver linje)*

	Aldri	Sjelden	Ofte	Alltid
Når jeg først har begynt å spise, kan det være vanskelig å stoppe	☐	☐	☐	☐
Jeg kaster opp etter at jeg har spist	☐	☐	☐	☐
Jeg bruker for mye tid til å tenke på mat	☐	☐	☐	☐
Jeg føler at maten kontrollerer livet mitt	☐	☐	☐	☐
Når jeg spiser, skjærer jeg maten opp i små biter	☐	☐	☐	☐
Jeg bruker lengre tid enn andre på et måltid	☐	☐	☐	☐
Andre mennesker synes at jeg er for tynn	☐	☐	☐	☐
Jeg føler at andre presser meg til å spise	☐	☐	☐	☐

7. Vil du si om deg selv at du er: *(Sett ett kryss)*

Svært tykk	Litt tykk	Omtrent som andre	Heller tynn	Svært tynn
☐	☐	☐	☐	☐

8. Prøver du å slanke deg? (*Sett ett kryss)*

☐ Nei, vekten min er passe ☐ Nei, men jeg trenger å slanke meg ☐ Ja
