# Supplementary material for: Maternal serum retinol, 25(OH)D and 1,25(OH)2D concentrations during pregnancy and peak bone mass and trabecular bone score in adult offspring at 26-year follow-up
Source: PLoS One. 2019 Sep 26;14(9):e0222712. doi: 10.1371/journal.pone.0222712 (PMC6762137; doi:10.1371/journal.pone.0222712)
Supplement: S12 File — (PDF) [file pone.0222712.s015.pdf]

OM MOTORIKK OG FYSISK AKTIVITET

1. I hvilken grad opplever du at du har motoriske vansker?

☐ Ikke i det hele tatt ☐ I liten grad ☐ I noen grad ☐ I stor grad ☐ I svært stor grad

2. I hvilken grad påvirker de motoriske vanskene aktiviteter i hverdagen?

☐ Ikke aktuelt (har ikke motoriske vansker)

I arbeid: ☐ Ikke i det hele tatt ☐ I liten grad ☐ I noen grad ☐ I stor grad ☐ I svært stor grad

I fritid: ☐ Ikke i det hele tatt ☐ I liten grad ☐ I noen grad ☐ I stor grad ☐ I svært stor grad

3. Vil du si at disse vanskene er hovedsakelig:

☐ Finmotoriske vansker ☐ Grovmotoriske vansker ☐ Begge deler

4. Har du fått hjelp for dine motoriske vansker? ☐ Nei ☐ Ja

5. Hvis ja, av hvem?

☐ fysioterapeut ☐ ergoterapeut ☐ fastlege ☐ helsesøster ☐ sykepleier ☐ PPT

Hva slags hjelp? ____

Opplevde du at dette hjalp? ☐ Nei ☐ Ja

6. Hvor fysisk anstrengende er arbeidet/studiet ditt?

☐ Jeg arbeider/studerer ikke

☐ Mitt arbeid/studie er hovedsakelig sittende arbeid og jeg beveger meg ikke mye i arbeidstiden (f.eks. en urmakers, radiomekanikers, industrisyerskes arbeid, kontorarbeid ved skrivebordet)

☐ Jeg beveger meg ganske mye i mitt arbeid/studie, men jeg trenger ikke løfte eller bære tunge gjenstander (f.eks. en arbeidsleders eller butikkekspeditørs arbeid, lett industriarbeid, kontorarbeid som krever at man beveger seg)

☐ Jeg er i arbeidet/studiet nødt til å gå og løfte mye eller ofte gå opp trapper eller bakker (f.eks. en snekkers eller vaktmesters arbeid, maskinverksted - eller liknende tyngre industriarbeid)

☐ Mitt arbeid/studie er tungt kroppsarbeid, hvor jeg er nødt til å løfte og bære tunge gjenstander, grave, skyfle eller hakke osv. (f.eks. skogsarbeid, tungt jordbruksarbeid, tungt bygge- eller industriarbeid)

7. Hvor mange timer i uka arbeider/studerer du? __ __ timer __ __ min

8. **Hvor mange minutter går, sykler eller beveger du deg på andre fysisk anstrengende måter under reise til og fra arbeid/studiested?** *Obs! Her regnes den sammenlagte tiden for reisen til og fra arbeid/studiested.*

- ☐ Jeg arbeider/studerer ikke, eller ferdes hele veien til arbeidet/studiestedet med motorkjøretøy
- ☐ Mindre enn 15 minutter per dag
- ☐ 15-29 minutter per dag
- ☐ 30-44 minutter per dag
- ☐ 45-59 minutter per dag
- ☐ 1 time eller mer per dag

9. **Hvor mange timer sitter du i gjennomsnitt på en hverdag?**

I arbeids-/studietiden på kontor eller liknende	__ __ t __ __ min
Hjemme foran tv, video eller data	__ __ t __ __ min
I kjøretøy	__ __ t __ __ min
Andre steder	__ __ t __ __ min

10. **Utenom skole/arbeid: Hvor mange dager i uka driver du idrett, eller mosjonerer du så mye at du blir andpusten og/eller svett?** *Regn ikke med mosjon under reise til/fra arbeid.*

- ☐ Ikke i det hele tatt
- ☐ Sjeldnere enn 1 gang i måneden
- ☐ 1-2 ganger i måneden
- ☐ Ca. 1 gang i uka
- ☐ 2-3 ganger i uka
- ☐ 4-5 ganger i uka
- ☐ Ca. daglig

11. **Hvor lenge driver du idrett, eller mosjonerer du vanligvis per gang på fritiden?**

- ☐ Jeg mosjonerer ikke på fritiden
- ☐ Mindre enn 30 minutter
- ☐ 30-59 minutter
- ☐ 1- <2 timer
- ☐ 2 timer eller mer

12. Hvor hardt driver du idrett, eller mosjonerer du vanligvis?
Sett kryss ved det alternativet som beskriver intensiteten best.

☐ Gange

☐ Veksler mellom gange og lett løping

☐ Lett løping (jogging)

☐ Energisk løping (intervalltrening)

13. På en skala fra 6 til 20, hvor 6 ikke er anstrengende og 20 er maksimalt anstrengende: Hvor tung er belastningen vanligvis når du driver idrett eller mosjonerer?
Nedenfor følger en forklaring og beskrivelse av belastningen ved noen av tallene.

☐ 6 Ingen anstrengelse. Du merker ikke noen anstrengelse i det hele tatt, for eksempel ingen muskeltretthet, ingen andpustenhet eller pusteplager

☐ 7 Meget, meget lett

☐ 8

☐ 9 Meget lett. Som å gå en kort tur i sitt eget tempo

☐ 10

☐ 11 Ganske lett

☐ 12

☐ 13 Litt anstrengende. Du kan fortsette uten større problemer

☐ 14

☐ 15 Anstrengende. Du er sliten, men kan likevel fortsette

☐ 16

☐ 17 Meget anstrengende. En veldig stor påkjenning. Du kan fortsette, men må ta i veldig hardt og føler deg svært sliten

☐ 18

☐ 19 Svært anstrengende. For de fleste mennesker tilsvarer dette den aller største anstrengelsen de noensinne har opplevd

☐ 20 Maksimalt anstrengende

14. Hva slags mosjon/idrett driver du med? Nevn alle aktiviteter:

____

____

15. Hvor mange minutter i gjennomsnitt per dag går, sykler eller beveger du på deg på annen måte på fritiden *(i hus- og hagearbeid, reparasjonsarbeid, rydding/vasking)?*
(Regn ikke med den tiden som du er aktiv i arbeidet, eller i reise til og fra arbeid, eller den tiden av fritiden som du driver idrett/mosjon)

☐ Mindre enn 15 minutter per dag

☐ 15-29 minutter per dag

☐ 30-44 minutter per dag

☐ 45-59 minutter per dag

☐ 1 time eller mer per dag

16. Har du vanligvis <u>minst 30 minutter</u> fysisk aktivitet daglig på arbeid og/eller fritida?

☐ Nei ☐ Ja

17. Hvordan anser du din fysiske form å være for tiden?

☐ Svært bra ☐ Ganske bra ☐ Passe ☐ Ganske dårlig ☐ Svært dårlig

18. Har du noen gang prøvd anabole steroider eller andre dopingmidler? *(Sett ett kryss)*

☐ Nei ☐ Ja

Hvis ja, hvor gammel var du første gang? _____ år gammel

19. Har du brukt anabole steroider eller andre dopingmidler siste 3 måneder?

☐ Nei ☐ Ja
