# Supplementary material for: Maternal serum retinol, 25(OH)D and 1,25(OH)2D concentrations during pregnancy and peak bone mass and trabecular bone score in adult offspring at 26-year follow-up
Source: PLoS One. 2019 Sep 26;14(9):e0222712. doi: 10.1371/journal.pone.0222712 (PMC6762137; doi:10.1371/journal.pone.0222712)
Supplement: S13 File — (PDF) [file pone.0222712.s016.pdf]

SPØRSMÅL FOR KVINNER

1. **Har du fast kjæreste?** ☐ Nei ☐ Nei, ikke nå, men før ☐ Ja

   Er du fornøyd med kjæreste-livet ditt? ☐ Nei ☐ Nei, ikke nå, men før ☐ Ja

2. **Hvor gammel var du da du fikk din første menstruasjon?** Jeg var ____ år og _____ måneder

3. **Har du regelmessig menstruasjon?** ☐ Nei ☐ Ja ____ uker mellom hver menstruasjon

4. **Hvor mange menstruasjoner har du hatt <u>de siste 12 månedene</u>?**_____ antall

5. **Hvor mange dager er det siden første dag i siste menstruasjon?** _____ dager

6. **Har du noen gang brukt p-piller?**

   ☐ Nei ☐ Ja, jeg har brukt det før ☐ Ja, jeg bruker det nå

   Hvis ja:
   Hvor gammel var du første gang du brukte p-piller? _______ år gammel

   Hvor lenge har du brukt p-piller i alt? _______ år til sammen

7. **Har du noen gang brukt noen av følgende prevensjonsmidler?**

	Nei	Ja, jeg har brukt det før	Ja, jeg bruker det nå
P-sprøyte / P- stav / Mini-pille	☐	☐	☐
P- ring / P-plaster	☐	☐	☐
Spiral uten hormoner	☐	☐	☐
Spiral med hormoner	☐	☐	☐
Pessar	☐	☐	☐
Kondom	☐	☐	☐
Avbrutt samleie	☐	☐	☐
Sterilisering	☐	☐ Ja, meg selv	☐ Ja, partner

8. **Har du noen gang hatt samleie?** ☐ Nei ☐ Ja

   Hvis ja, hvor gammel var du første gang ______ år

9. **Har du noen gang brukt angrepille?** ☐ Nei ☐ Ja ☐ Flere ganger

   Hvis ja, hvor gammel var du første gang ______ år

10. Har du noen gang vært gravid? ☐ Nei ☐ Ja

	1. gang	2. gang	3. gang
Hvis ja, hvor gammel var du da dette skjedde?	______ år	______ år	______ år
Ønsket du denne graviditeten? *(Kryss for hver graviditet)*	☐ Nei<br>☐ Ja<br>☐ Vet ikke	☐ Nei<br>☐ Ja<br>☐ Vet ikke	☐ Nei<br>☐ Ja<br>☐ Vet ikke
Ble det utført abort? Sett kryss bare hvis JA	☐	☐	☐

11. Er du gravid nå? ☐ Nei ☐ Ja Hvis ja, antall uker: ______

12. Har du barn? ☐ Nei ☐ Ja Hvis ja, oppgi antall: ______

Hvor gammel var du da du ble mor første gang? Jeg var ____ år og _____ måneder

Barn 1: Fødselsvekt: ______ g Lengde: _____ cm Svangerskapslengde:____ uker ____dager

☐ Født for tidlig (>3 uker før termin) ☐ Født til termin (uke 37 - 42) ☐ Født etter uke 42

Barn 2: Fødselsvekt: ______ g Lengde: _____ cm Svangerskapslengde:____ uker ____ dager

☐ Født for tidlig (>3 uker før termin) ☐ Født til termin (uke 37 – 42) ☐ Født etter uke 42

Barn 3: Fødselsvekt: ______ g Lengde: _____ cm Svangerskapslengde:____ uker ____ dager

☐ Født for tidlig (>3 uker før termin) ☐ Født til termin (uke 37 - 42) ☐ Født etter uke 42

13. Hvor lenge er det siden siste fødsel? ____ år og _____ måneder

14. Har du fått menstruasjon etter siste fødsel? ☐ Nei ☐ Ja

15. Ammer du nå? ☐ Nei ☐ Ja
