# Supplementary material for: Maternal serum retinol, 25(OH)D and 1,25(OH)2D concentrations during pregnancy and peak bone mass and trabecular bone score in adult offspring at 26-year follow-up
Source: PLoS One. 2019 Sep 26;14(9):e0222712. doi: 10.1371/journal.pone.0222712 (PMC6762137; doi:10.1371/journal.pone.0222712)
Supplement: S14 File — (PDF) [file pone.0222712.s017.pdf]

# SPØRSMÅL FOR MENN

**1. Har du fast kjæreste?** ☐ Nei ☐ Nei, ikke nå, men før ☐ Ja

Er du fornøyd med kjæreste-livet ditt? ☐ Nei ☐ Nei, ikke nå, men før ☐ Ja

**2. Har du noen gang hatt samleie?** ☐ Nei ☐ Ja

Hvis ja, hvor gammel var du første gang ______ år

**3. Har en kvinne noen gang blitt gravid med deg?** ☐ Nei ☐ Ja

| | 1. gang | 2. gang | 3. gang |
|---|---|---|---|
| Hvis ja, hvor gammel var du da dette skjedde? | ______ år | ______ år | ______ år |
| Ønsket du denne graviditeten? *(Kryss for hver graviditet)* | ☐ Nei<br>☐ Ja<br>☐ Vet ikke | ☐ Nei<br>☐ Ja<br>☐ Vet ikke | ☐ Nei<br>☐ Ja<br>☐ Vet ikke |
| Ble det utført abort? Sett kryss bare hvis JA | ☐ | ☐ | ☐ |

**4. Har du barn?** ☐ Nei ☐ Ja Hvis ja, oppgi antall: ______

Hvor gammel var du da du ble far første gang? Jeg var ____ år og _____ måneder

Barn 1: Fødselsvekt: ______ g Lengde: _____ cm Svangerskapslengde:____ uker ____dager

☐ Født for tidlig (>3 uker før termin) ☐ Født til termin (uke 37 - 42) ☐ Født etter uke 42

Barn 2: Fødselsvekt: ______ g Lengde: _____ cm Svangerskapslengde:____ uker ____ dager

☐ Født for tidlig (>3 uker før termin) ☐ Født til termin (uke 37 – 42) ☐ Født etter uke 42

Barn 3: Fødselsvekt: ______ g Lengde: _____ cm Svangerskapslengde:____ uker ____ dager

☐ Født for tidlig (>3 uker før termin) ☐ Født til termin (uke 37 - 42) ☐ Født etter uke 42
